# Positional determination of the carbon–carbon double bonds in unsaturated fatty acids mediated by solvent plasmatization using LC–MS

**DOI:** 10.1038/s41598-020-69833-y

**Published:** 2020-07-31

**Authors:** Shigeo Takashima, Kayoko Toyoshi, Takuhei Yamamoto, Nobuyuki Shimozawa

**Affiliations:** 1 Division of Genomics Research, Life Science Research Center, Gifu University, 1-1 Yanagido, Gifu City, Gifu 501-1193 Japan; 2 United Graduate School of Drug Discovery and Medical Information Sciences, Gifu University, Gifu, 501-1193 Japan; 3 Gifu Pharmaceutical University, Gifu, 501-1196 Japan

**Keywords:** Lipids, Metabolomics, Analytical biochemistry, Mass spectrometry

## Abstract

Fatty acids (FAs) are the central components of life: they constitute biological membranes in the form of lipid, act as signaling molecules, and are used as energy sources. FAs are classified according to their chain lengths and the number and position of carbon–carbon double bond, and their physiological character is largely defined by these structural properties. Determination of the precise structural properties is crucial for characterizing FAs, but pinpointing the exact position of carbon–carbon double bond in FA molecules is challenging. Herein, a new analytical method is reported for determining the double bond position of mono- and poly-unsaturated FAs using liquid chromatography-mass spectrometry (LC–MS) coupled with solvent plasmatization. With the aid of plasma on ESI capillary, epoxidation or peroxidation of carbon–carbon double bond in FAs is facilitated. Subsequently, molecular fragmentation occurs at or beside the epoxidized or peroxidized double bond via collision-induced dissociation (CID), and the position of the double bond is elucidated. In this method, FAs are separated by LC, modified by plasma, fragmented via CID, and detected using a time-of-flight mass spectrometer in a seamless manner such that the FA composition in a mixture can be determined. Our method enables thorough characterization of FA species by distinguishing multiple isomers, and therefore can uncover the true diversity of FAs for their application in food, health, and medical sciences.

## Introduction

Fatty acids (FAs) are the central components of living organisms as they are structural components of phospholipids and sphingolipids that constitute biological membranes, can be converted into physiologically active signal molecules (i.e. eicosanoids and lysophospholipids), and serve as energy sources^1–5^. Their chemical characteristics are primarily determined by their carbon chain length as well as the number and position of carbon–carbon double bonds. Saturated fatty acids (SFAs) do not contain carbon–carbon double bonds and their chemical stability is suitable for cellular energy storage. In contrast, unsaturated FAs contain one or more carbon–carbon double bonds and are relatively unstable compared to SFAs, especially when multiple double bonds are present. Unsaturated FAs with a single carbon–carbon double bond are referred to as mono-unsaturated fatty acids (MUFAs), while those with multiple carbon–carbon double bonds are collectively called poly-unsaturated fatty acids (PUFAs). Unsaturated FAs can be further categorized into several groups, such as *ω*-3, *ω*-6, and *ω*-9 family FAs, where each family is distinguished by the position of the first double bond in relation to the omega carbon. The number and position of double bonds in FAs dictate their biological activities and are considered to be important subjects for human health^2,6,7^.

Gas chromatography-mass spectrometry (GC–MS) is traditionally used to analyze FAs. To determine the position of carbon–carbon double bonds, derivatization of unsaturated FAs at the double bonds, such as with dimethyl disulfide as a derivatization agent, is often applied. The derivatized unsaturated FAs are ionized using electron ionization (EI) in GC–MS. The high energy of EI enables the cleavage of the modified bond to elucidate the diagnostic fragment ions from which the position of the double bond can be determined^8–11^. Liquid chromatography-mass spectrometry (LC–MS) complements GC–MS in many applications including FA analysis, and it is adequate for very-long-chain FAs (FAs with 22 or more carbons) that have poor volatility in GC–MS^12^. The determination of the position of carbon–carbon double bond in unsaturated FAs using LC–MS, however, remains challenging. Electrospray ionization (ESI) and atmospheric pressure chemical ionization (APCI), both often used in LC–MS, are the methods involving soft ionization; so, fragmentation does not occur effectively by itself. Subsequently, collision-induced dissociation (CID) is used to fragment the ionized FAs. However, it usually does not provide the informative fragment ions to determine the double bond position. To generate the informative fragment ions by CID, modification of FAs on carbon–carbon double bonds or the terminal carboxyl group is required. For example, acetonitrile-related adducts of FA methyl esters produce diagnostic fragment ions from which the double bond position can be determined^13,14^. Epoxidation of the double bond of FAs by low-temperature plasma successfully produces the informative diagnostic fragment ions from free FAs and lipid-conjugated FAs after CID fragmentation^15,16^. Epoxidation was also facilitated by use of catalytic agent^17–19^. Paper-spray ionization followed by CID also successfully epoxidize FAs and produce the same type of diagnostic fragment ions to identify the position of carbon–carbon double bonds^20^. Paternó–Büchi reaction with acetone also modifies the double bond, which subsequently produces diagnostic fragment ions via CID^21–24^. Derivatization with *N*-(4-aminomethylphenyl)pyridinium (AMPP) at the terminal carboxy group also provides the informative fragment ions for carbon–carbon double bond^25,26^. With these methods one can deduce the exact position of the carbon–carbon double bond of unsaturated FAs.

We previously reported a method for analyzing a range of FA species in biological samples in a single assay by using reverse-phase LC–MS^12,27^, where FAs were distinguished by their chain lengths and number of double bonds. Using this method, approximately 50 FA species could be distinguished in control human fibroblasts and > 100 species in patient fibroblasts with Zellweger syndrome (ZS), wherein the biosynthesis of peroxisomes is impaired and FA metabolism is altered. Although we could estimate and compare the approximate positions of the double bonds among FA isomers, the exact position of the double bonds could not be determined.

In the present study, we established a method that can determine the exact position of the carbon–carbon double bonds in unsaturated FAs by using conventional LC–MS without specialized setups. Herein, we induced epoxidation and peroxidation of the carbon–carbon double bond of unsaturated FAs facilitated by solvent plasmatization as corona discharge. Subsequent CID produced the diagnostic fragment ions for determining the double bond position. Peroxidation, but not epoxidation, of PUFA was found to be required for producing the informative diagnostic fragment ions. We applied our method to analyze FAs from biological samples. In combination with our previous method, the current method enables full characterization of a FA species and thorough profiling of the FA composition in biological samples or in food resources.

## Materials and methods

### Reagents

The FA standards used in this study were as follows: *cis*-6-octadecenoic acid (C18:1 *ω*-12, petroselinic acid; Sigma-Aldrich, St. Louis, MO, USA, #P8750), *cis*-9-octadecenoic acid (C18:1 *ω*-9, oleic acid; Cayman Chemical, Ann Arbor, MI, USA, #90260), *cis*-11-octadecenoic acid (C18:1 *ω*-7, *cis*-vaccenic acid; Matreya LLC, State College, PA, USA, #1266), ^13^C-UL-oleic acid (^13^C-labeled oleic acid; Martek Isotopes, Olney, MD, USA), all *cis*-9, 12, 15-octadecatrienoic acid (C18:3 *ω*-3, α-linolenic acid; Cayman Chemical, #90210), all *cis*-6, 9, 12-octadecatrienoic acid (C18:3 *ω*-6, γ-linolenic acid; Cayman Chemical, #90220), all *cis*-5, 11, 14-eicosatrienoic acid (20:3 *ω*-6, sciadonic acid; Cayman Chemical, #10009999), all *cis*-8, 11, 14-eicosatrienoic acid (20:3 *ω*-6, dihomo-γ-linolenic acid; Cayman Chemical, #90230), all *cis*-5, 8, 11, 14, 17-eicosapentaenoic acid (20:5 *ω*-3, EPA; Cayman Chemical, #90110.1), all *cis*-5, 8, 11, 14-eicosatetraenoic acid (20:4 *ω*-6, arachidonic acid, ARA; Cayman Chemical, #90010), all *cis*-4, 10, 13, 16-docosatetraenoic acid (C22:4 *ω*-6, *cis*-4 DTA, Cayman Chemical, #10007289), all *cis*-4, 7, 10, 13, 16, 19-docosahexaenoic acid (22:6 *ω*-3, DHA, Tokyo Chemical Industry/TCI, Tokyo, Japan, #D2226), *trans*-9-hexadecenoic acid (9*E*1-C16:1, palmitelaidic acid; Nu-Chek-Prep, Inc., Elysian, MN, USA, #U-41A), *trans*-11-octadecenoic acid (11*E*-C18:1, *trans*-vaccenic acid; Nu-Chek-Prep, #U-49-A). The FA standards were dissolved in a solution containing two volumes of chloroform and one volume of methanol with 0.05% (w/v) of 2,6-di-*t*-butyl-*p*-cresol (butylated hydroxytoluene; Nacalai Tesque, Kyoto, Japan, #11421-92) and stored at − 20 °C until use. Other reagents used were: Deuterium oxide (D_2_O, FUJIFILM Wako Pure Chemical Corp., Osaka, Japan, #049-34242), Water-^18^O (H_2_^18^O, Taiyo Nippon Sanso Corp., Tokyo, Japan, #F03-0027), high-performance liquid chromatography (HPLC)-grade *tert*-butyl methyl ether (*t*-BME, Sigma-Aldrich, #34875), HPLC-grade acetonitrile (FUJIFILM Wako, #019-08631), HPLC-grade acetone (Wako, #014-08681), HPLC-grade 1 mM ammonium acetate (FUJIFILM Wako, #018-21041), and 10% ammonia solution (FUJIFILM Wako, #013-17505).

### Liquid chromatography-mass spectrometry

A Waters Acquity ultra-performance liquid chromatography system equipped with an auto sampler and reverse-phase column with thermal control was used. A Waters BEH C_8_ column (2.1 × 50 mm, particle size 1.7 µm, pore size 130 Å, #186002877) preceded by a Waters BEH C_8_ VanGuard Pre-Column (2.1 × 5 mm, particle size 1.7 µm, pore size 130 Å, #186003978) was used. Aqueous mobile phase A consisted of purified water containing 1 mM (0.0077% w/v) ammonium acetate and 5.78 mM (0.01% w/v) ammonia. The organic mobile phase B was 100% acetonitrile. The flow rate was 0.1 mL/min and the following linear gradient was applied (indicated content of mobile phase B): 0–50 min, 20–95% linear increase; 50–52.5 min, hold at 95%; 52.5–55 min, hold at 20%. Mass spectrometry was performed using a Waters XevoQTof MS system, which is a time-of-flight mass spectrometer preceded by a quadrupole and collision chamber. The mobile phase was plasmatized by charging high voltage on the ESI capillary to cause corona discharge. The samples were applied to MS from the LC system or via direct infusion channel without LC separation. The MS settings were as follows: capillary voltage on negative ESI, 1.0 kV for normal MS assay and 3.6–4.2 kV for plasmatization; sampling cone, 56 (arbitrary value); extraction cone, 4.0 (arbitrary value); source temperature, 125 °C; desolvation temperature, 350 °C; cone gas flow, 60 L/h; and desolvation gas flow, 1,000 L/h. Argon gas was used for collision-induced dissociation (CID). We removed lockspray baffle because it interferes stable plasma generation at the ESI tip so that lock mass was not used throughout the analysis. The obtained data were analyzed by Waters Masslynx software and ACD ChemSketch was used to draw the chemical structures.

### Direct infusion assay

For direct infusion assay, the FA standards were dissolved in 100% acetonitrile and directly introduced in MS via an infusion port and mixed with LC solvents A and B before infusion. The same MS settings as above were used. In some experiments, the FA standards were dissolved in a mixed solution of H_2_^18^O and acetonitrile or D_2_O and acetonitrile, and were analyzed without mixing with LC solvent.

### Extraction of fatty acids from human fibroblasts

Total FAs were purified from control human skin fibroblasts, NB1RGB, obtained from the National Bio-Resource Project (NBRP) of the Ministry of Education, Culture, Sports, Science, and Technology (MEXT), and from fibroblasts of a patient with ZS with *PEX2* mutation (F-12 line)^12^. A pellet of approximately 1 × 10^6^ cells was dissolved in 400 µL of acetonitrile and 50 µL of 5 M hydrochloric acid in a glass tube. The sample was lysed by vortexing for 1 min and incubated at 100 ℃ for 1 h in an oil bath. After cooling to room temperature (approximately 20 ℃), 800 µL of *t*-BME, 100 µL of methanol containing ^13^C-labeled oleic acid (internal standard; 1 µg/mL) and 400 µL of purified water were added, and vortexed for 1 min. Phase separation was achieved by a centrifugation at 200 × g for 5 min, and the upper organic phase was collected. Subsequently, 800 µL of water was added and the sample was vortexed again for 1 min. After phase separation at 200 × g for 5 min, the upper organic phase containing FAs was collected and placed in a glass sample vial with a polytetrafluoroethylene (PTFE)-lined cap (GL Sciences, Tokyo, Japan, #1030-51023 and #1030-45260). The organic phase was evaporated under a stream of nitrogen gas and the recovered FAs were reconstituted in 100 µL of acetone and subjected to LC–MS analysis.

### Ethics

This study was approved by the Ethical Committee of the Graduate School of Medicine, Gifu University (permission number: 29-286). For the use of a clinical sample obtained from an infant patient, written informed consent was obtained from the patient's parents. All experiments in this manuscript were conducted according to the guidelines and regulations provided by Gifu University.

## Results and discussion

### Solvent plasmatization facilitates epoxidation and peroxidation of unsaturated fatty acids for double bond position determination

**Figure 1:**
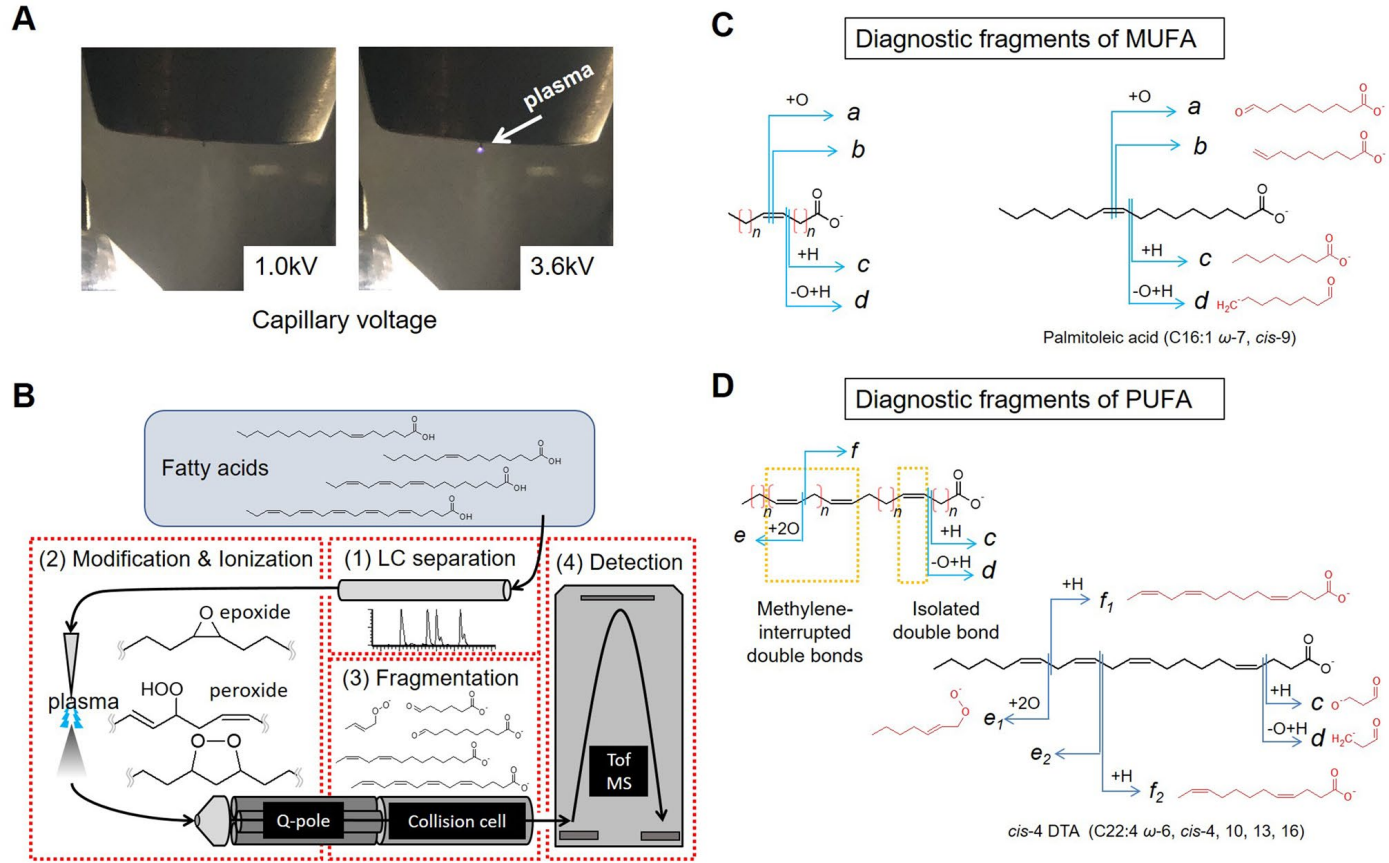
Summary of the method to determine the position of carbon–carbon double bonds in unsaturated FAs using LC–MS. (**A**) Plasmatization of the LC solvent on ESI probe at different electrical voltages. At a normal voltage (left, 1.0 kV), no plasmatization occurs, whereas plasmatization occurs at high voltage (right, 3.6 kV) at the tip of the ESI capillary (arrow). (**B**) A schematic of the LC–plasma–ESI–MS assay developed in this study. Q-pole: quadrupole, Tof MS: time-of-flight mass spectrometer. (**C**, **D**) Fragmentation patterns of a representative MUFA (**C**) and PUFA (**D**). Generalized fragmentation patterns are shown on the left and real examples are shown on the right. Diagnostic fragments (fragment *a* to fragment *f*) are indicated with expected molecular structures.

**Figure 2:**
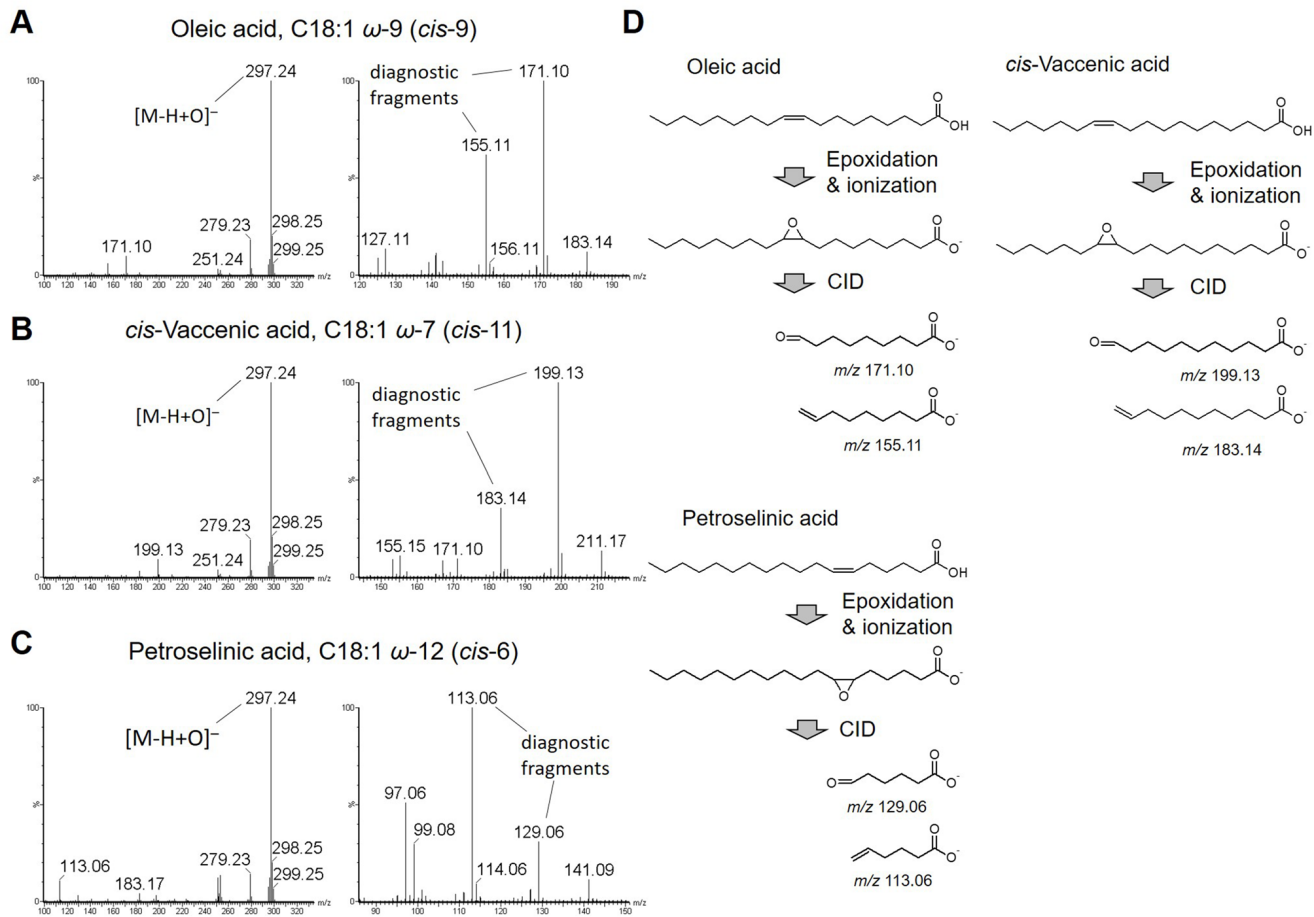
Structural determination of octadecenoic acids (C18:1). (**A**–**C**) Analysis of oleic acid (**A**), *cis*-vaccenic acid (**B**), and petroselinic acid (**C**) using a direct infusion assay. The parent ion ([M–H + O]^–^, *m/z* 297.24; marked in the left panels) and diagnostic fragment ions (right panels) of three C18:1 species are shown. (**D**) Flow of the diagnostic fragment formation. Throughout the manuscript, *y*-axis of the graphs indicates relative intensity and *x*-axis indicates either *m/z* (in spectral charts) or retention time (in mass chromatograms).

Zhao et al. reported that epoxidation of unsaturated FAs by low-temperature plasma and subsequent fragmentation using ESI–MS can determine the position of carbon–carbon double bond^15^. We tried a similar approach using LC–ESI–MS and found that unsaturated FAs can be epoxidized and peroxidized by plasmatization of the LC solvent. When an excessively high electric voltage was applied at the tip of the ESI capillary, plasmatization of the solvent occurred as corona discharge (Fig. 1A). Epoxidation of oleic acid (C18:1 *ω*-9, *cis*-9) was first attempted with direct infusion assay (100–200 pmol/min with 0.1 mL/min of solvent; isocratic 50% B). At a normal capillary voltage on negative ESI (1.0 kV), ionized oleic acid ([M–H]^–^, *m/z* 281.25) was detected (Supplementary Fig. S1A). When a high voltage (≥ 3.4 kV) was applied to the ESI capillary, plasmatization of the solvent was induced at the capillary tip, and simultaneously, the epoxidized oleic acid ([M–H + O]^–^, *m/z* 297.24) was detected in the mass spectrum (Fig. 2A, left panel; Fig. S1B). The epoxidized oleic acid molecule was fragmented by CID and produced fragment ions at *m/z* 171.10 and 155.11 (Fig. 2A, right panel; Fig. 2D), consisting of the fragments containing the alpha carbon (hereafter “alpha fragments”), from which the double bond position can be determined as shown in the previous studies^15,20^.

In addition to the epoxidized form, peroxidized form ([M–H + 2O^–^, *m/z* 313.24]) (Fig. S1B) was also generated on solvent plasmatizaton as a side product. This side reaction always occurred irrespective of given electric voltages so that was unable to eliminated. However, we found that the peroxidized form can also be used for determining the double bond positions as shown in the following section. When methanol and isopropanol were used for the solvent, same reaction happened to form epoxide and peroxide, though use of isopropanol unstabilized plasma formation at the tip of ESI capillary. In this study, we chose to use acetonitrile because it has better elution capability on LC for FA analysis.

The same protocol was attempted using *cis*-vaccenic acid (C18:1 *ω*-7, *cis*-11) and petroselinic acid (C18:1 *ω*-12, *cis*-6), which have the same chain length and number of double bonds, but different double bond positions compared to those in oleic acid. Following epoxidation and CID, similar results were obtained while producing the diagnostic fragments for the determination of the double bond position (Fig. 2B–D). Thus, solvent plasmatization as corona discharge was found to be useful for the epoxidation of FAs and this method is referred to as plasma–ESI–MS.

We next applied plasma–ESI–MS with LC analysis. The entire scheme of the analysis, LC–plasma–ESI–MS, is depicted in Fig. 1B; and the diagnostic fragment ions for determining the position of carbon–carbon double bond in MUFAs and PUFAs found in the above and following experiments are shown in Fig. 1C,D.

### Determination of the double bond position of MUFAs by LC–MS

We analyzed octadecenoic acids (C18:1 FAs) with LC-plasma-ESI–MS. In our previous study^27^, the proximity of the double bond to the omega carbon was found to be the major determinant of retention time of unsaturated FAs in reverse-phase LC. *Cis*-vaccenic acid (*ω*-7) elutes from the LC column first, oleic acid (*ω*-9) elutes second, and petroselinic acid (*ω*-12) elutes last^27^. Here, this was also confirmed by determining the exact position of the double bond. C18:1 FAs were injected independently and simultaneously as a mixed sample. In the independent injection, each C18:1 species yielded a sharp peak at a distinct retention time (Fig. S2A). The epoxidized form of each C18:1 species was detected, and by CID, the diagnostic fragment ions were produced (Figs. S2B,C; fragment *a* and fragment *b* in Fig. 1C). In the mixture injection, peaks were imperfectly separated (Fig. S3A–C). The epoxidized form was detected for all peaks. Upon CID, the fragment ions of the epoxidized *cis*-vaccenic acid were observed at the earliest retention time at the first half of the first peak (Fig. S3A), the fragments of epoxidized oleic acid were observed at the second half of the first peak (Fig. S3B), and the fragments of epoxidized petroselinic acid were observed in the second peak (Fig. S3C). Epoxidized *cis*-vaccenic acid showed fragments at *m/z* 199.13 and 183.14 with a peak position at 17.82 min (Fig. S3A). The fragments for the epoxidized oleic acid, *m/z* 171.10 and 155.11, showed peaks at 18.24 and 18.14 min, respectively (Fig. S3B). The fragments for the epoxidized petroselinic acid, *m/z* 129.06 and 113.06, showed a peak at 18.66 min (Fig. S3C). These time differences confirmed the elution order of C18:1 FA species: *cis*-vaccenic acid, oleic acid, and then petroselinic acid. The above data confirmed our previous observations that showed the order of elution timing of unsaturated FAs correlated with the proximity of the double bond to the omega carbon.

As shown in the previous section, FAs can be peroxidized by the solvent plasmatization. We found that the fragmentation spectra of the peroxidized FAs are also informative for determining the position of the double bonds. On solvent plasmatiozation, peroxidized oleic acid (*m/z* 313.24) was found simultaneously with the epoxidized oleic acid as a side reaction, though the intensity is lower than the epoxidized form (Fig. S1B). Upon CID, peroxidized oleic acid produced the same fragment ions as the epoxidized form (Fig. S4A,B), such as the alpha fragments at *m/z* 171.10 and 155.11. Additional fragment ions were also observed at *m/z* 143.11 and 127.11 corresponding to the alpha fragments with and without terminal oxygen (fragment *c* and fragment *d* in Fig. 1C). Minor fragment ions such as peroxidized alpha fragment at *m/z* 201.11, and the fragment at *m/z* 141.13 corresponding to the omega carbon-side fragment (omega fragment) were also detected (Fig. S4A,B). Similar results were obtained for the analysis of peroxidized *cis*-vaccenic acid and petroselinic acid (Fig. S4C–F). These additional fragments yielded positional information of the double bond, and similar fragments were informative for determining the position of double bonds in PUFAs, which will be discussed in later sections.

### *Trans* FAs had same fragmentation patterns with *cis* FAs but longer retention time

We examined fragmentation patterns of *trans* FAs. Palmitelaidic acid (C16:1 *trans*-9) and *trans-*vaccenic acid (C18:1 *trans*-11; Figure S5B) were analyzed and compared with corresponding *cis* counterparts (palmitoleic acid and *cis*-vaccenic acid, respectively). The *trans* FAs yielded the same fragmentation patterns with their *cis* counterparts, including diagnostic fragment ions, when their epoxide or peroxide forms were fragmented (Fig. S5C and data not shown). There were no *cis* FA or *trans* FA specific fragments detected, therefore the *cis*/*trans* conformation could not be determined by their fragmentation patterns. However, the *trans* FAs could be distinguished from *cis* FAs on mass chromatogram; the *trans* FAs had longer retention times compared to the *cis-* FA counterparts (Fig. S5A,B). Therefore, this property can be used to distinguish *trans* FAs in analytical samples when comparable *cis* FAs are available.

### Determination of the position of double bonds in PUFAs

We next examined if the plasma-mediated modification of PUFAs could yield any informative fragment spectra for determining the position of carbon–carbon double bonds. Two types of octadecatrienoic acids (C18:3), α-linolenic acid (C18:3 *ω*-3, *cis*-9, 12, 15) and γ-linolenic acid (C18:3 *ω*-6, *cis*-6, 9, 12) were first examined by LC–plasma–ESI–MS. The form of epoxide was detected with predicted *m/z* value (*m/z* 293.21). Additionally, we found two types of peroxide with mass difference of two units (*m/z* 309.21 and 311.22) (Figs. S6A and S7A). The first one with *m/z* 309.21 was considered as regular peroxide as found in MUFA above, and the second one with *m/z* of 311.22 was predicted as cyclic peroxide (Figs. S6B and S7B), which the similar form was reported previously^28^.

**Figure 3:**
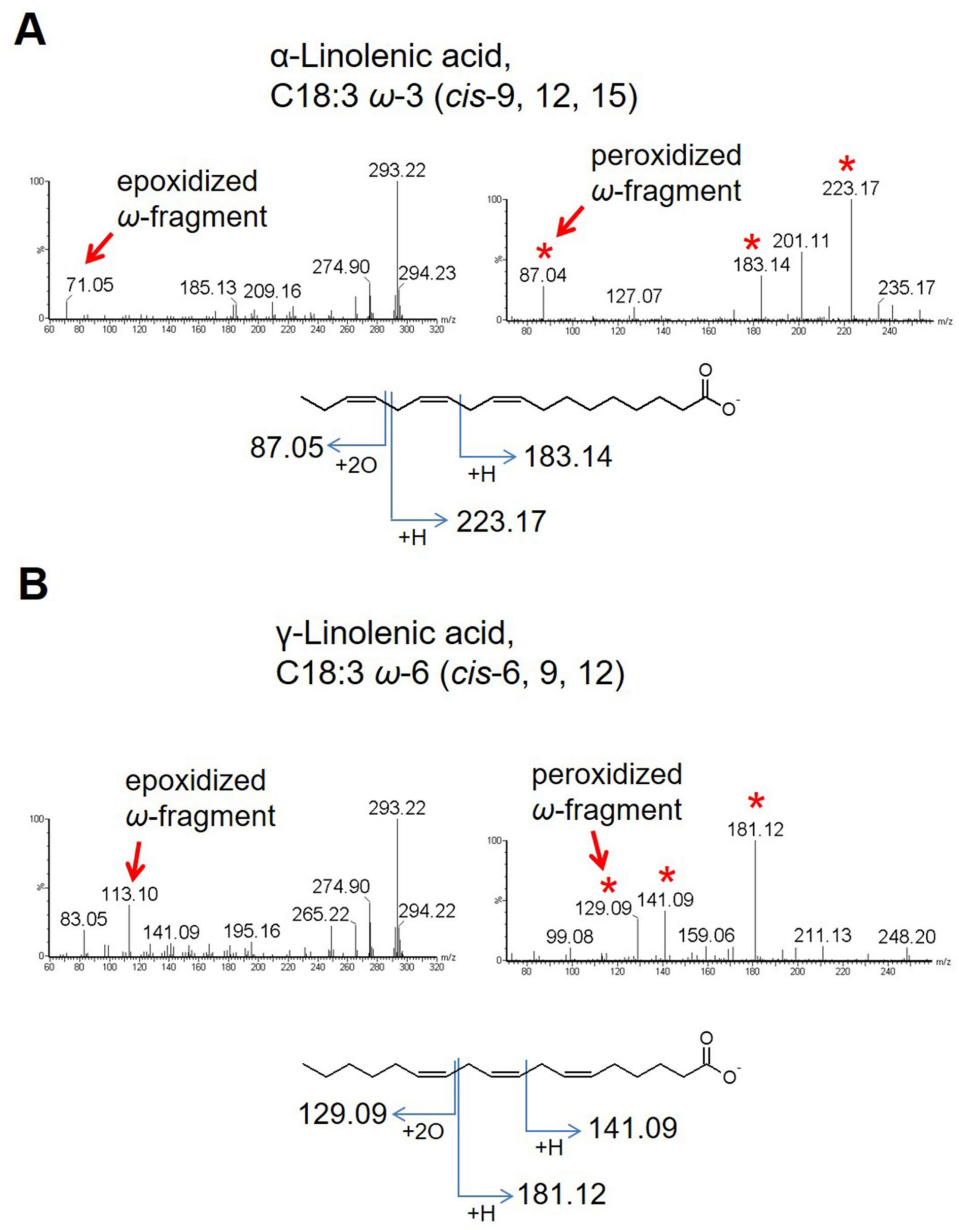
Structural determination of C18:3 FAs. (**A**, **B**) The epoxide (left panels) or cyclic peroxide (right panels) of α-linolenic acid and γ-linolenic acid were fragmented. The epoxidized and peroxidized fragments at the proximal double bond to the omega carbon are indicated by arrows and the diagnostic fragment ions from peroxidized C18:3 FAs are indicated by asterisks. The graphical fragmentation patterns for diagnostic fragment ions after peroxidation are also shown.

We compared the fragmentation patterns of each epoxidized/peroxidized form. When epoxidized form was subjected to CID, highly prominent fragment spectra at *m/z* 71.05 in α-linolenic acid and *m/z* 113.10 in γ-linolenic acid (Fig. 3A,B, left panels) were yielded. These spectra corresponded to the omega fragments epoxidized at the proximal double bond to the omega carbon. These types of fragmentation spectra always appeared in extremely high intensities in PUFAs and can be used for determining the double bond that is closest to the omega carbon for their categorization into *ω*-n families. The two types of peroxide were examined separately. When regular peroxide of α-linolenic acid and γ-linolenic acid were fragmented, ions at *m/z* 87.05 and 129.09, respectively, were detected in high intensities, and were peroxidized omega fragments at the proximal double bond to the omega carbon (Fig. 3A,B, right panels). The alpha fragments at *m/z* 223.17 and 181.12, which are the counterpart fragments of the above omega fragments, were also detected in high intensities (Fig. 3A,B, right panels). From the omega and alpha fragments, the positions of the first and second double bonds can be determined. Similar alpha fragments were observed at the second and third double bonds (*m/z* 183.14 and 141.09 for α- and γ-linolenic acid, respectively; Fig. 3A,B). The fragmentation yielding this type of alpha fragments only effectively occurs when the neighboring double bonds are separated by a single methylene group (hereafter referred to as “methylene-interrupted double bonds”; Fig. 1D) and not by multiple methylene groups (hereafter referred to as “isolated double bond”; Fig. 1D). In addition, there were multiple minor fragment ions that could support the positional information of double bonds as shown in Figs. S6D and S7D. When the cyclic peroxides of α-linolenic acid and γ-linolenic acid were fragmented, they yielded the same diagnostic fragment ions with regular peroxide but higher intensities (Figs. S6E, S7E). The same minor fragment ions with regular peroxide were also found with a few exceptions. The details of the fragmentation patterns of each peroxide were shown in Figs. S6 and S7. From these diagnostic fragments, the positions of all the three double bonds can be determined. Because both types of PUFA peroxide generate the diagnostic fragment ions, they were simultaneously fragmented to yield higher signals of diagnostic fragment ions for quantitative purposes, while independently fragmented for qualitative purposes in this study.

Two types of eicosatrienoic acid (C20:3), sciadonic acid (C20:3 *ω*-6 *cis*-5, 11, 14) and dihomo-γ-linolenic acid (C20:3 *ω*-6 *cis*-8, 11, 14), were examined. Dihomo-γ-linolenic acid contains three methylene-interrupted double bonds, whereas sciadonic acid contains two methylene-interrupted double bonds and one isolated double bond (Fig. S8C). From the fragmentation pattern of the peroxidized form, dihomo-γ-linolenic acid showed a similar fragmentation pattern as that of the C18:3 fatty acids described above (Fig. S8A,C; the fragment spectra of cyclic peroxide are shown). In contrast, sciadonic acid yielded the same fragmentation pattern only from its methylene-interrupted double bonds (*cis*-11 and *cis*-14 double bonds) (Fig. S8B,C). The isolated double bond at *cis*-5 position behaved in a similar manner as a MUFA double bond, producing common alpha fragments (Figs. S4 and S8B,C), enabling the determination of the position of the isolated double bond. The same fragmentation pattern was observed with the isolated double bond of *cis*-4 DTA, which contains three methylene-interrupted double bonds and an isolated double bond (see below). Therefore, the double bond positions in PUFAs can be determined by the fragment ions specific to the methylene-interrupted and isolated double bonds.

**Figure 4:**
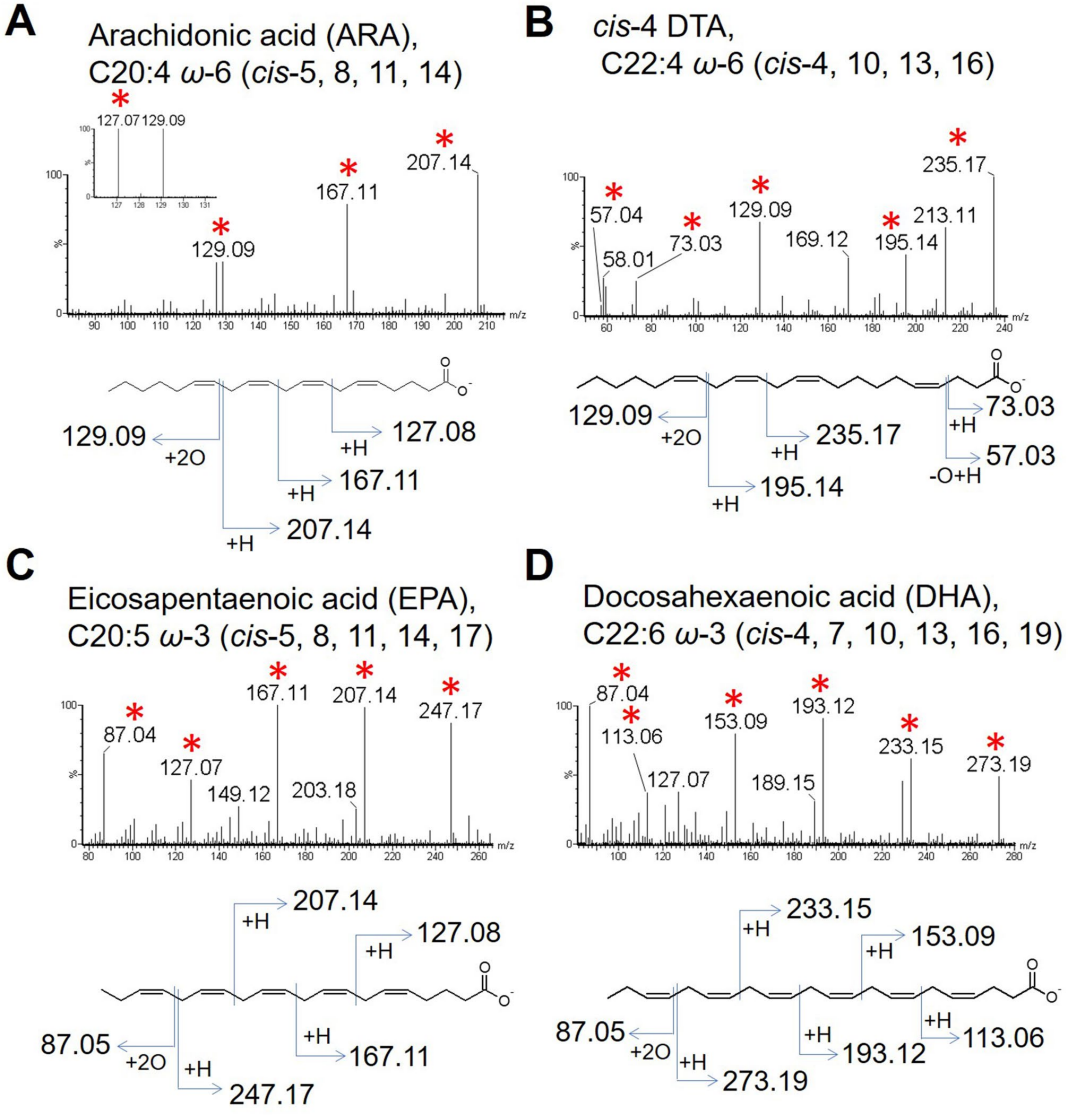
Structural determination of the PUFAs with four or more double bonds. (**A**, **B**) Fragmentation patterns of cyclic peroxide of arachidonic acid (ARA) (**A**) and *cis*-4 DTA (**B**) are shown. (**C**, **D**) Fragmentation patterns of highly unsaturated FA (cyclic peroxide), eicosapentaenoic acid (EPA) containing five double bonds (**C**) and docosahexaenoic acid (DHA) with six double bonds (**D**) are shown. The diagnostic fragment ions are indicated by asterisks.

In PUFAs with four or more double bonds, diagnostic alpha and omega fragments were more prominent, while the minor fragments decreased in intensity. As the PUFAs shown above, the methylene-interrupted double bonds in ARA (C20:4 *ω*-6, *cis*-5, 8, 11, 14) (Fig. 4A), *cis*-4 DTA (C22:4 *ω*-6, *cis*-4, 10, 13, 16) (Fig. 4B), EPA (C20:5 *ω*-3, *cis*-5, 8, 11, 14, 17) (Fig. 4C), and DHA (22:6 *ω*-3, *cis*-4, 7, 10, 13, 16, 19) (Fig. 4D) yielded the same type of diagnostic omega and alpha-fragments (fragments *e* and *f*s in Fig. 1D). The isolated double bond in *cis*-4 DTA can be positioned using the common alpha fragments with MUFA (*m/z* 73.03 and 57.03; fragments *c* and *d* in Fig. 1D). The above data demonstrate that the double bond position of a wide range of PUFA species can be determined by peroxidation and CID fragmentation with the common fragmentation criteria.

### Analytical performance

The analytical performance of this method was evaluated. Standard solutions containing single FA species, such as palmitoleic acid (C16:1, *cis*-9), *cis*-vaccenic acid (C18:1, *cis*-11), dihomo-γ-linolenic acid (C20:3, *cis*-8, 11, 14), *cis*-4 DTA (C22:4 *cis*-4, 10, 13, 16), and EPA (C20:5 *cis*-5, 8, 11, 14, 17), were quantified. Calibration functions were calculated for all the diagnostic fragment ions from both types of peroxide, and good linearity was confirmed in both MUFA and PUFA species (Table 1, Fig. S9). We used the diagnostic fragment ion giving the least intensity to calculate the limit of detection (LOD) and limit of quantification (LOQ) of the corresponding FA species. The calculated LOD was 6–15 μM, and LOQ was 19–45 μM among examined FA species (Table 1).

**Table 1:** Analytical performances of MUFA and PUFA species.

Fatty acid standard	Diagnostic fragmentions (*m/z*)^a^	Calibration function	Correlation coefficient (R^2^)	LOD (microM)^b^	LOQ (microM)^b^	Calibration range
Palmitoleic acid	155.11	y = 32.698x + 56.772	0.9990	7.30	22.12	2–200 μM
C16:1 *cis*-9	171.10	y = 60.945x + 65.594	0.9997			
*cis*-Vaccenic acid	183.14	y = 53.336x + 151.16	0.9947	8.49	25.72	5–100 μM
C18:1 *cis*-9	199.13	y = 147.06x + 397.99	0.9960			
Dihomo-γ-linolenic acid	129.09	y = 36.723x + 179.24	0.9964	14.36	43.50	5–200 μM
C20:3 *cis*-8, 11, 14	169.12	y = 51.625x + 212.14	0.9961			
	209.15	y = 118.42x + 590.78	0.9954			
*cis*-4 DTA	57.03	y = 18.442x + 95.653	0.9973	6.56	19.89	5–100 μM
C22:4 *cis*-4, 10, 13	73.03	y = 24.578x + 111.09	0.9951			
	129.09	y = 55.475x + 230.71	0.9948			
	195.14	y = 31.22x + 104.74	0.9980			
	235.17	y = 73.223x + 261.94	0.9971			
EPA	87.05	y = 14.684x + 22.201	0.9982			
C20:5 *cis*-5, 8, 11, 14, 17	127.08	y = 11.486x + 10.306	0.9986	8.47	25.66	5–200 μM
	167.11	y = 20.692x + 2.6985	0.9993			
	207.14	y = 19.227x − 12.693	0.9993			
	247.17	y = 16.67x + 1.6052	0.9990			

*RSD* residual standard deviation.

^a^Diagnostic fragment ions with least intensities are underlined.

^b^LOD and LOQ were calculated as 3.3 × RSD/slope and 10 × RSD/slope, respectively, based on the calibration function.

We also evaluated the intraday (n = 5) and interday (n = 5) reproducibilities of diagnostic fragment ions using standard solutions of *cis*-vaccenic acid and γ-linolenic acid at two different concentrations (10 μM and 100 μM). The intraday precision, expressed as relative standard deviation (RSD), was less than 10% for most of the fragments (Table 2). The interday precision of RSD without internal standard (IS; ^13^C18:1 *cis*-9) was high; however, it improved upon normalization using IS (Table 2).

**Table 2:** Intraday and Interday precisions associated with this method.

Fatty acid standard	Concentration	Diagnostic fragments (*m/z*)	Intraday RSD (%) without I.S. (%)	Intraday RSD (%) with I.S.^a^ (%)	Interday RSD (%) without I.S. (%)	Interday RSD (%) with I.S.^a^ (%)
*cis*-Vaccenic acid	10 μM	183.14	5.9	6.2	26.5	8.3
C18:1 *cis*-9		199.13	6.9	5.4	26.4	4.7
	100 μM	183.14	4.2	7.3	28.0	9.2
		199.13	3.6	8.0	28.4	5.6
γ-Linolenic acid	10 μM	129.09	1.3	4.0	35.7	13.7
C18:3 *cis*-6, 9, 12		141.09	8.3	9.8	31.2	6.1
		181.12	11.8	12.7	32.4	6.7
	100 μM	129.09	5.7	10.9	31.6	15.0
		141.09	3.9	9.0	30.3	12.4
		181.12	4.7	10.4	30.1	12.6

^a^Fragment ions of the internal standard, ^13^C-labeled oleic acid, was used for normalization.

Relative quantification was evaluated with mixtures of FA isomers of MUFA (oleic acid and *cis*-vaccenic acid), and PUFA (α-linolenic acid γ-linolenic acid), with several different ratios of the two isomers (Table 3). The relative quantity of the two isomers showed good linearity in the range from 0.1 to 2.0. The above data indicate that the relative abundance of FA isomers can be quantified using this method.

**Table 3:** Analytical performances in relative quantification.

First isomer	Second isomer	Corresponding fragments of 2nd/1st isomers (*m/z*)	Calibration function	R2	Ratio range (2nd/1st isomer)
Oleic acid	*cis*-Vaccenic acid	183.14/155.11	y = 0.5744x + 0.3371	0.9982	0.1–2.0
C18:1 *cis*-9	C18:1 *cis*-11	199.13/171.10	y = 0.7582x + 0.0724	0.9954	
100 μM	10–200 μM				
α-Linolenic acid	γ-Linolenic acid	129.09/87.05	y = 0.5368x + 0.0217	0.9995	0.1–2.0
C18:3 *cis*-9, 12, 15	C18:3 *cis*-6, 9, 12	141.09/183.14	y = 0.4983x + 0.1484	0.9876	
100 μM	10–200 μM	181.12/223.17	y = 0.5328x + 0.0592	0.9988	

### Ionization and fragmentation mechanisms

**Figure 5:**
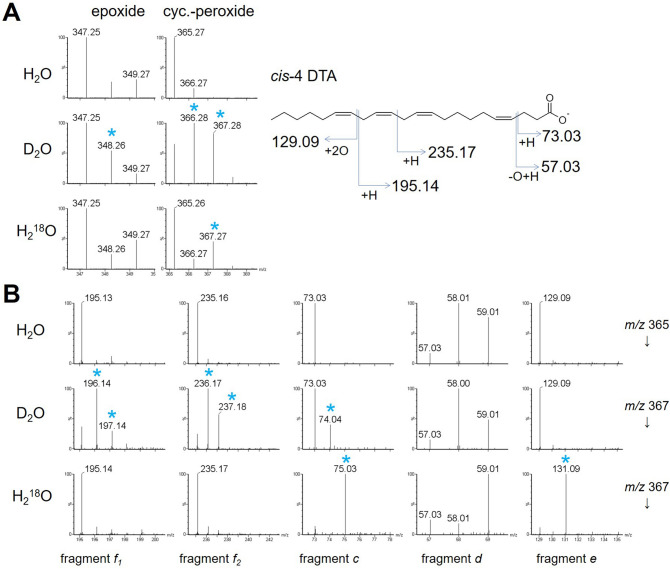
Mechanistic background of peroxide formation and fragmentation. (**A**) Mass spectra of the cyclic peroxide of *cis*-4 DTA. There was no mass increase in the cyclic peroxide with H_2_^18^O; however, there was a mass increase by one unit and two units with D_2_O (asterisk) or by two units with H_2_^18^O. (**B**) Diagnostic fragment ions of *cis*-4 DTA. The alpha fragments (i.e. m/z 195.14, 235.17, 73.03, and 57.03) show mass increase (asterisks) when D_2_O is used as the solvent, in contrast to the omega fragment (*m/z* 129.09) that does not show mass increase. When H_2_^18^O was used there was mass increases in the omega fragment as well as in fragment *c.* The fragments with m/z 58.01, showing decrease in H_2_^18^O, is a non-target fragment.

The analytical mechanisms of this method were also studied. To elucidate the origin of oxygen in the epoxide and peroxide groups of the FAs formed by the plasma-ESI, we used ^18^O-containing water (H_2_^18^O) as a solvent. *Cis*-4 DTA was dissolved in a 1:1 solution of H_2_^18^O and acetonitrile, and was examined by direct infusion assay (additives like ammonium acetate and ammonia solution were not supplemented to eliminate any contaminant of regular water). Epoxide formed by plasma-ESI had the same *m/z* values as those obtained using regular water (Fig. 5A), indicating that the oxygen in epoxide group did not came from the solvent, but probably from the surrounding air as in the case of a previous report^15^. In contrast, cyclic peroxide of *cis*-4 DTA had higher *m/z* value with two units (2 Da). The fragment ions containing peroxide group also had 2 Da increase (Fig. 5B). These results suggest that one of the two oxygen atoms in the cyclic peroxide group comes from the solvent and the other oxygen atom comes from the surrounding air.

We next used deuterium oxide (D_2_O) as a solvent to reveal the origin of the added hydrogen in the peroxide group and in some fragment ions (*i.e.* diagnostic fragment ions *c*, *d*, *e*, and *f*; see Fig. 1C,D) by the same direct infusion assay. We found peroxidized *cis*-4 DTA and *cis*-vaccenic acid had increased *m/z* values, indicating that the solvent was the origin of the hydrogen in the peroxide group (Fig. 5 and Fig. S10, respectively). The diagnostic alpha fragment ions (diagnostic fragment ions *c*, *d*, and *f*) also had increased *m/z* values by 1 Da, again indicating that the hydrogen added to the fragments was derived from the solvent. On the other hand, the omega fragment (diagnostic fragment *e*) did not have increased *m/z* value. Taken together, these data indicate that the hydrogens are included in the diagnostic fragment ions in the plasma-ESI method.

### Analysis of unsaturated fatty acids from human fibroblasts

**Figure 6:**
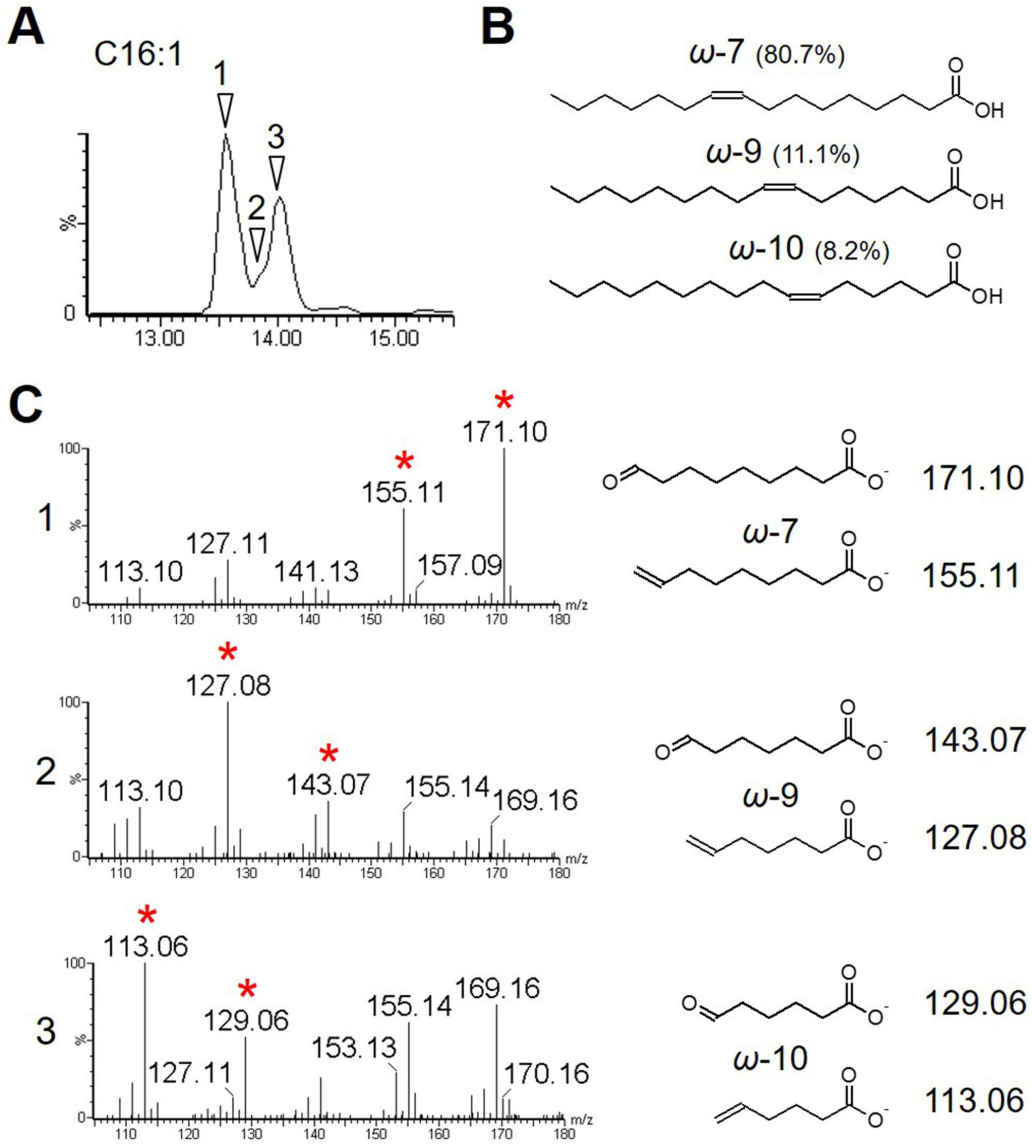
Identification of multiple C16:1 FA isomers in human fibroblasts. (**A**) Mass chromatogram of C16:1 (*m/z* 253.22). The time points where each C16:1 isomer was identified are indicated by arrowheads. (**B**) Detected C16:1 isomers from the analysis. Three C16:1 isomers with double bonds at *ω*-7, *ω*-9, and *ω*-10 were discovered and their abundance ratio are shown. *cis*/*trans* isomerism is not considered. (**C**) Tandem mass spectra and diagnostic fragment structure of each C16:1 isomer at the indicated time points in (**A**) are shown and diagnostic fragment ions are indicated by asterisks.

The developed method was subsequently applied to biological samples. FAs were extracted from human fibroblasts (NB1RGB) via acid hydrolysis and separated using a reverse-phase gradient LC (see “Materials and methods”). The quadrupole was set to introduce epoxidized MUFA species (epoxi-MUFAs), including C16:1, C18:1, C20:1, C22:1, C24:1, and C26:1, in separate time windows. The introduced epoxi-MUFAs were subsequently fragmented by CID. The entire ions ranging from *m/z* 100 to 1,000 were also scanned and recorded simultaneously in another channel to detect the non-epoxidized FAs. As reported previously^27^, multiple peaks can be observed for some of the MUFAs in their mass chromatograms (Fig. 6A; Figs. S12A, S13A), which may represent FA isomers with different double bond positions. This observation was confirmed by characterizing the isomers in each peak. In the mass chromatogram of C16:1, two peaks were observed with incomplete base separation. Upon CID fragmentation, three C16:1 isomers (Fig. 6) were observed. In the first peak, *ω*-7 isomer yielding fragment ions at *m/z* 155.11 and 171.10 was detected. At the beginning area of the second peak, *ω*-9 isomer was observed yielding fragments at *m/z* 143.07 and 127.08, while *ω*-10 isomer yielding fragments at *m/z* 113.06 and 129.06 was found throughout most of the second peak. The retention order of the isomers followed previous observations where the FAs with more proximal double bonds to the omega carbon eluted faster than those with more distal double bonds.

From the C18:1 assay, four isomers were detected (Figure S11), with the most abundant being oleic acid (C18:1 *ω*-9), which yielded a fragment ion pair at *m/z* 171.10 and 155.11. The other C18:1 isomers were *ω*-7 (producing a fragment ion pair at *m/z* 199.13 and 183.14), *ω*-10 (*m/z* 157.09 and 141.09), and *ω*-12 (*m/z* 129.05 and 113.06). *ω*-7, *ω*-9, and *ω*-10 isomers were observed in the first major peak, while *ω*-12 isomer was detected in the minor second peak (Fig. S11). Comparing the retention times of the isomers, the elution order from the earliest to the latest was *ω*-7, *ω*-9, *ω*-10, and *ω*-12 isomers (Fig. S11). Even though four isomers of C18:1 were detected, the high abundance of prospective oleic acid (*ω*-9 isomer) embedded the other isomers within a single large peak, highlighting the importance of isomer segregation. Similarly, multiple isomers were observed in the assays of C20:1 to C26:1. The isomers with their diagnostic fragment ions are shown in Figs. S12–S15. Though the isomeric variety of longer FAs are not well known, the isomers of C16:1 and C18:1 found in our study were consistent with previous reports^15,16,19,20,26,29^. The double bond position of MUFAs from biological samples, thus, were confirmed by this method.

**Figure 7:**
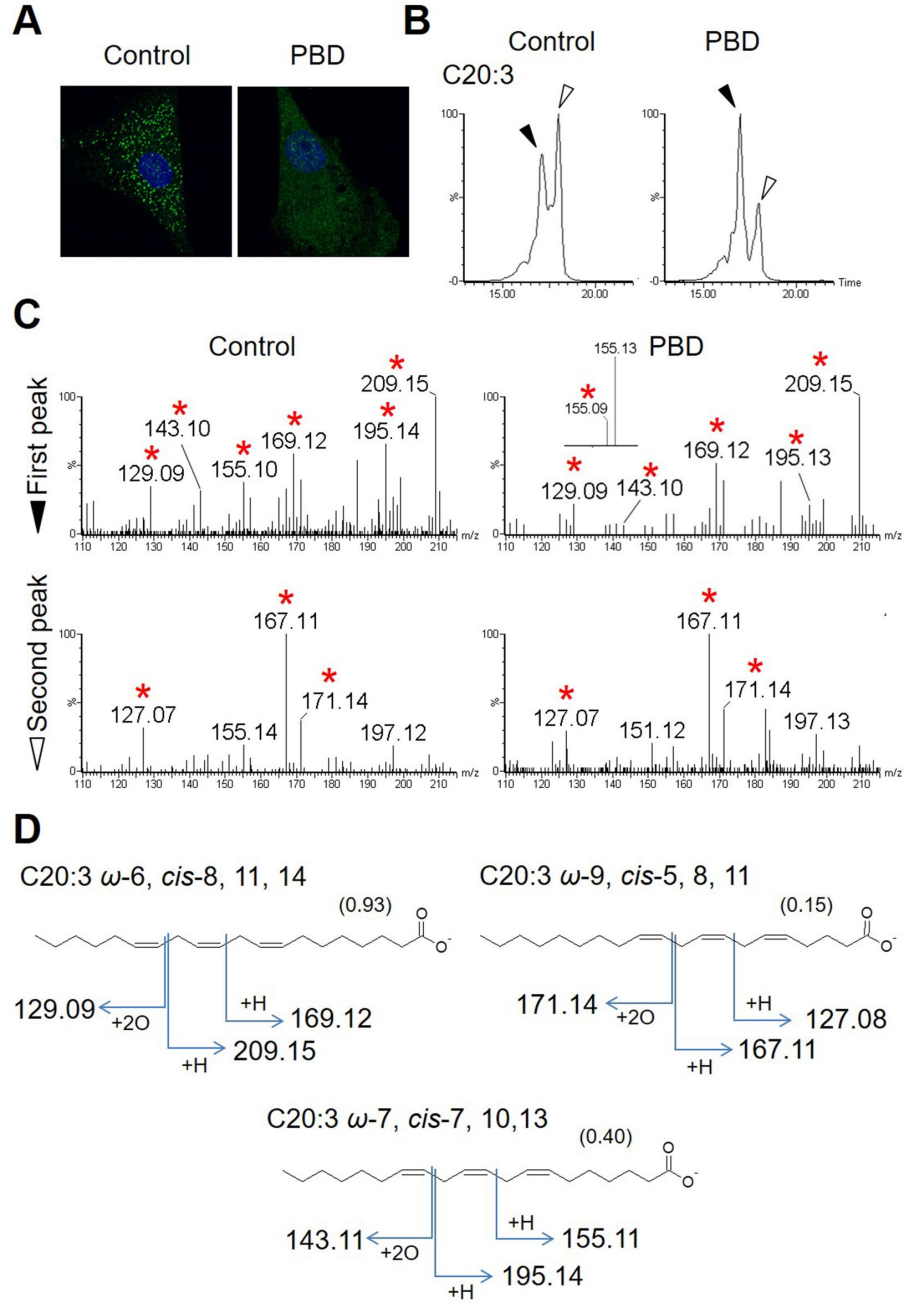
Identification and comparison of the C20:3 PUFA species between control fibroblasts and fibroblasts from a ZS patient. (**A**) Anti-catalase staining (green) of the fibroblasts. The nuclei were stained with DAPI in blue. Vesicular peroxisomes are visible only in the control fibroblast. (**B**) Mass chromatogram of C20:3 (*m/z* 305.25) where the peak tops are indicated by black (first peak) and white (second peak) arrowheads. (**C**) Tandem mass spectra at each peak where the diagnostic fragment spectra are indicated by asterisks. (**D**) Chemical structures and diagnostic fragmentation patterns of the detected C20:3 species are shown. Their relative abundances in PBD fibroblasts against control are shown in parentheses.

Finally, PUFA isomers from a clinical sample were analyzed. FAs were extracted from fibroblasts of a human patient with Zellweger syndrome (ZS) and from control fibroblasts (F-12 and NB1RGB, respectively)^12^. In ZS, the biosynthesis of peroxisomes is impaired (Fig. 7A) and the FA composition fluctuates due to metabolic failures in peroxisomes. In our previous study, multiple peaks were obtained for C20:3 FA upon LC separation, where each peak was predicted to be a distinct C20:3 isomer, although the exact double bond position remained undetermined^27^. By analyzing the FAs from the fibroblast samples, multiple peaks for C20:3 were confirmed again (Fig. 7B). Peroxidation of C20:3 followed by CID fragmentation indicated that the first peak included a C20:3 isomer with the double bonds at *cis*-8, 11, and 14 positions in ZS and control fibroblasts (Fig. 7B–D). Moreover, an additional isomer with double bonds at *cis*-7, 10, and 13 positions was found in both samples but reduced in ZS fibroblasts (Fig. 7C,D, Fig. S16). The second peak corresponded to only a single isomer with double bonds at *cis*-5, 8, and 11 positions found in both samples but again reduced in ZS fibroblasts (Fig. 7C,D, Fig. S16). These results confirmed our previous prediction about multiple peaks of C20:3 in control and ZS fibroblasts^27^. These data indicate that the PUFA isomers in biological samples can be distinguished by the double bond position. This is the first example that determined the double bond positions of PUFA isomers showing differential abundances in ZS samples, highlighting the usefulness of the method reported herein for medical applications.

## Conclusions

Determination of the positions and number of double bonds, as well as the chain length, is crucial for understanding the precise physiological functions of each FA species. Many methods have been established for the positional determination of double bonds by modifying the target FAs at the double bond positions or the terminal carboxyl group to yield diagnostic fragment ions^8–11,13–26^. CID-mediated fragmentation on ESI–MS or EI-mediated fragmentation on GC–MS of unsaturated FAs cannot always produce informative product ions to determine the double bond positions, especially in case of PUFAs with many double bonds. Moreover, the derivatization of FAs would affect their chromatographic retention time, compromising the interpretation of chromatographic results. Herein, a method of post-column epoxidation and peroxidation of unsaturated FAs using corona discharge plasmatization of chromatographic solvent is reported. This method is advantageous for samples containing mixed FAs, such as biological samples. The post-column epoxidation/peroxidation does not compromise the LC, so the chain length and number of double bonds in each FA in the mixture can be easily determined from the LC results^27^. Similar approach was reported recently, where FA epoxidation was facilitated by low voltage on nano-ESI sprayer^30^, although there are significant technical differences such as the existence of hydrochloric acid and the polarity of ESI capillary.

The drawback of our method, presently, is its low sensitivity, mainly attributable to the poor epoxidation/peroxidation efficiency. Moreover, we could not separately induce epoxidation or peroxidation on requirement therefore cannot eliminate unwanted derivatives from the reaction. The reported LOQ of FAs in regular LC–MS or GC–MS analysis is around nanomolar order so that our method is currently only suitable for FAs with high abundance. However, our method can be used as a supporting method with regular LC–MS/GC–MS analyses to show the isomeric heterogeneity of detected FA species. Moreover, our method only requires conventional reagents and equipment for LC–MS therefore easy to try with.

For the improvement, several approaches are conceivable. Because we used a pre-equipped setup of conventional ESI, the development of a specialized setup may improve the sensitivity for detecting FA species with low abundance. Alternatively, addition of an epoxidizing/peroxidizing agent to the solvent would improve the sensitivity of this method. In addition, post-column modification of target molecules with solvent plasmatization can be applied to other types of molecules for structural analysis. In conclusion, the method reported herein enabled a thorough characterization of FA species, i.e. chain length, number of double bonds, and position of double bonds, and can aid for many applications.

## Supplementary information

Supplementary Figures.
