# Supplementary material for: Positional determination of the carbon–carbon double bonds in unsaturated fatty acids mediated by solvent plasmatization using LC–MS
Source: Sci Rep. 2020 Jul 31;10:12988. doi: 10.1038/s41598-020-69833-y (PMC7395107; doi:10.1038/s41598-020-69833-y)
Supplement: Supplementary file 1 — Supplementary Figures. [file 41598_2020_69833_MOESM1_ESM.docx]

**Supplementary Information**

**Positional determination of the carbon-carbon double bonds in unsaturated fatty acids mediated by solvent plasmatization using LC-MS.**

Shigeo Takashima^1,2,*^, Kayoko Toyoshi^1^, Takuhei Yamamoto^3^, and Nobuyuki Shimozawa^1,2^

^1^ Division of Genomics Research, Life Science Research Center, Gifu University, Gifu 501-1193, Japan

^2^ United Graduate School of Drug Discovery and Medical Information Sciences, Gifu University, Gifu 501-1193, Japan

^3^ Gifu Pharmaceutical University, Gifu 501-1196, Japan

**
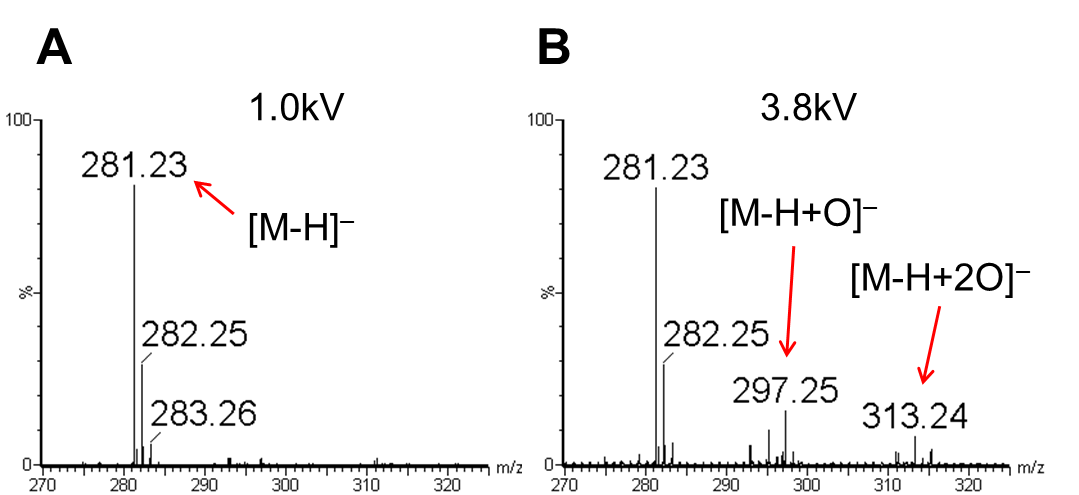
**

**Figure S1.** Plasma-facilitated modification of oleic acid in direct infusion assay. (A, B) Mass chromatogram of oleic acid with different capillary voltage. At l.0 kv (A), oleic acid was detected as deprotonated monovalent anion ([M-H]^–^; calculated *m/z* of 281.25). At high voltage (*e.g.* 3.8 kv, B), epoxidized, deprotonated anion ([M-H+O]^–^, *m/z* 297.24) and peroxidized, deprotonated anion ([M-H+2O]^–^, *m/z* 313.24) were detected simultaneously.

**
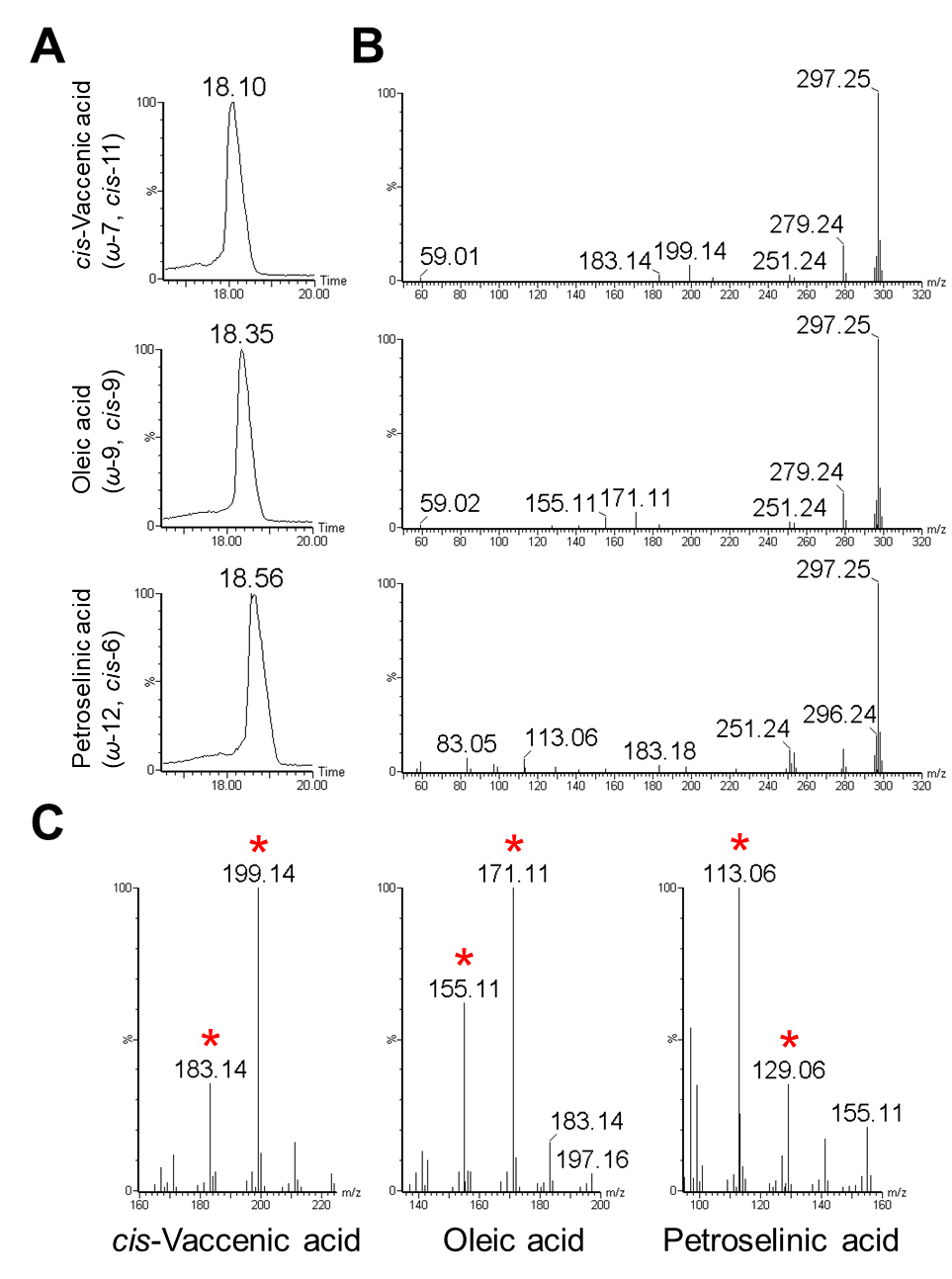
**

**Figure S2.** Structure determination of C18:1 FAs by LC-plasma-ESI-MS. (A) Mass chromatogram of oleic acid, *cis*-vaccenic acid, and petroselinic acid (*m/z* 281.25). Retention times are provided at the peak top. (B, C) Fragment spectra of the three FAs show the fragmentation pattern similar to the direct infusion assay (Figure 2). Diagnostic ions are indicated by asterisks in (C).

**
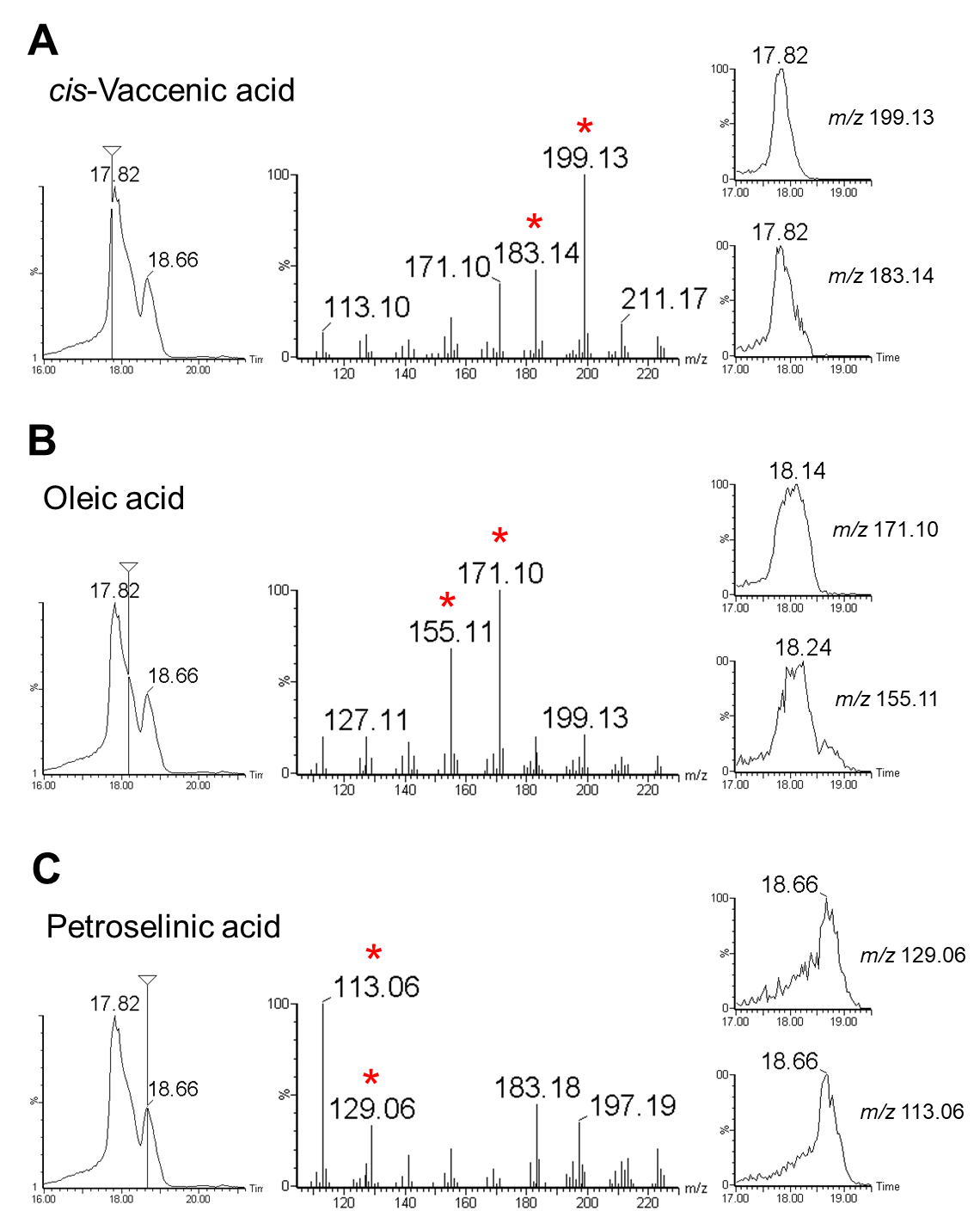
Figure S3.** Determination of the position of double bonds in C18:1 FAs using LC-plasma-ESI-MS. (A–C) Oleic acid, *cis*-vaccenic acid, and petroselinic acid were simultaneously injected and analyzed via LC-MS with plasma-mediated epoxidation. Total ion chromatogram from the MS/MS assay, with peak top time is shown in the left panels. The time points where the fragment spectra were extracted are indicated with vertical bars. The extracted spectra are shown in the middle panels and diagnostic fragment ions are indicated by asterisks. The tandem mass chromatograms of the diagnostic fragment ions are shown in the right panels with the peak top time indicated.


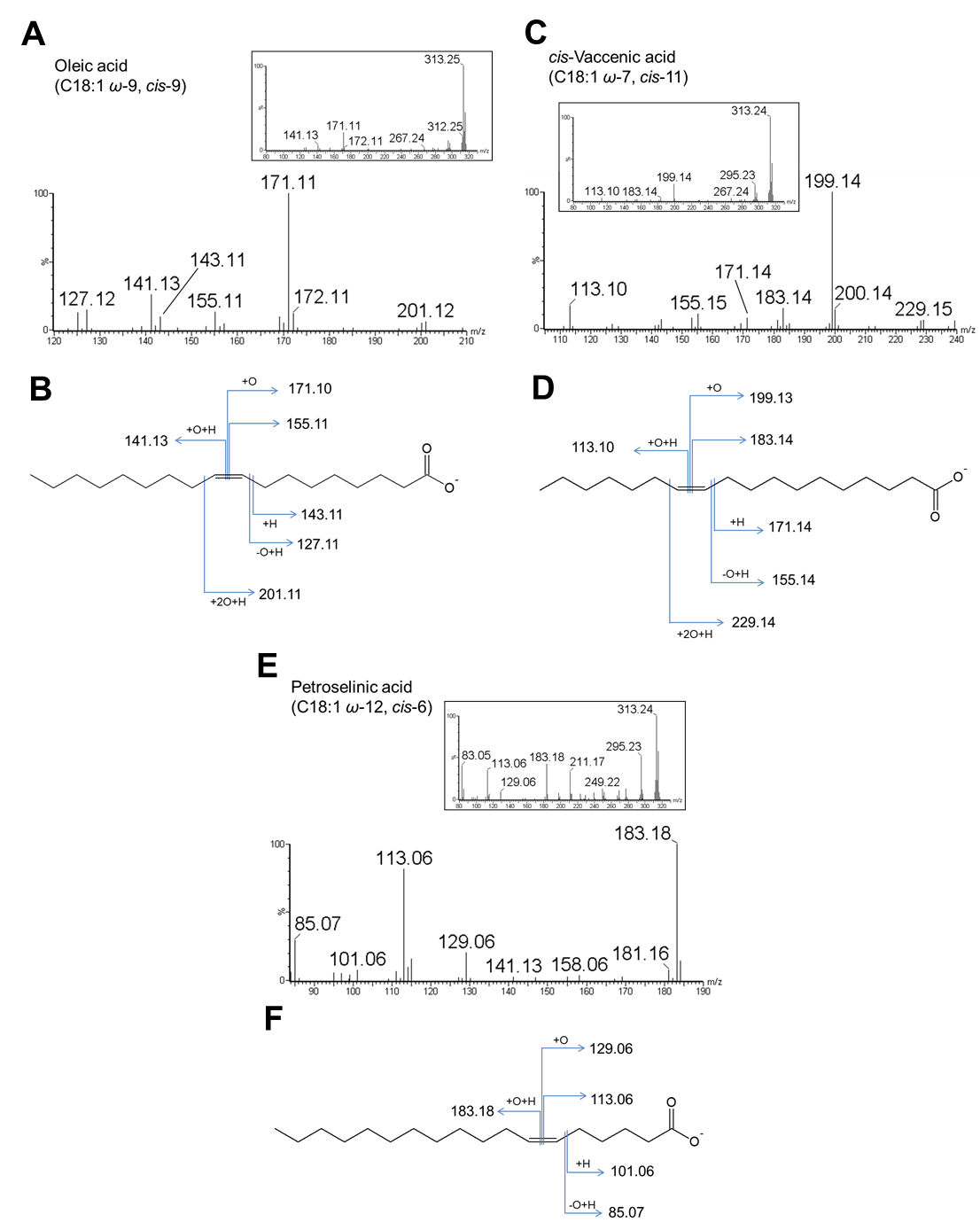


**Figure S4.** Fragmentation of C18:1 FAs. (A–F) Detailed fragment patterns of the peroxidized C18:1 FA species (*m/z* 313.24), such as oleic acid (A, B), *cis*-vaccenic acid (C, D), and petroselinic acid (E, F).


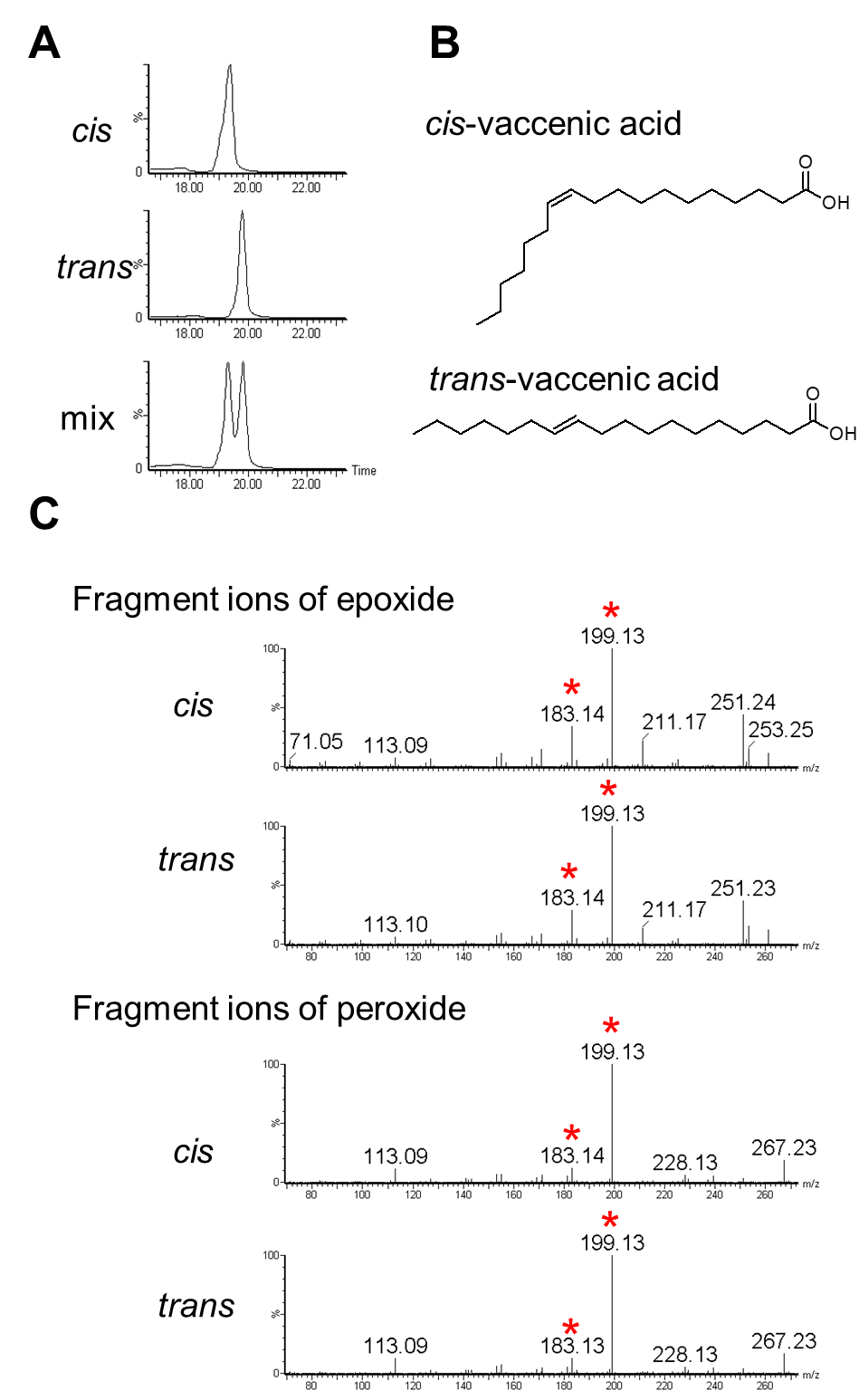


**Figure S5.** Comparison of *cis* and *trans* FAs. (A) Mass chromatogram of *cis*-vaccenic acid (top), *trans*-vaccenic acid (middle), and their mixture (bottom) are shown. (B) The structure of *cis-* and *trans-* vaccenic acids. (C) Fragment spectra of epoxide and peroxide of each isomer are shown. Diagnostic fragment ions are marked by asterisks.

**
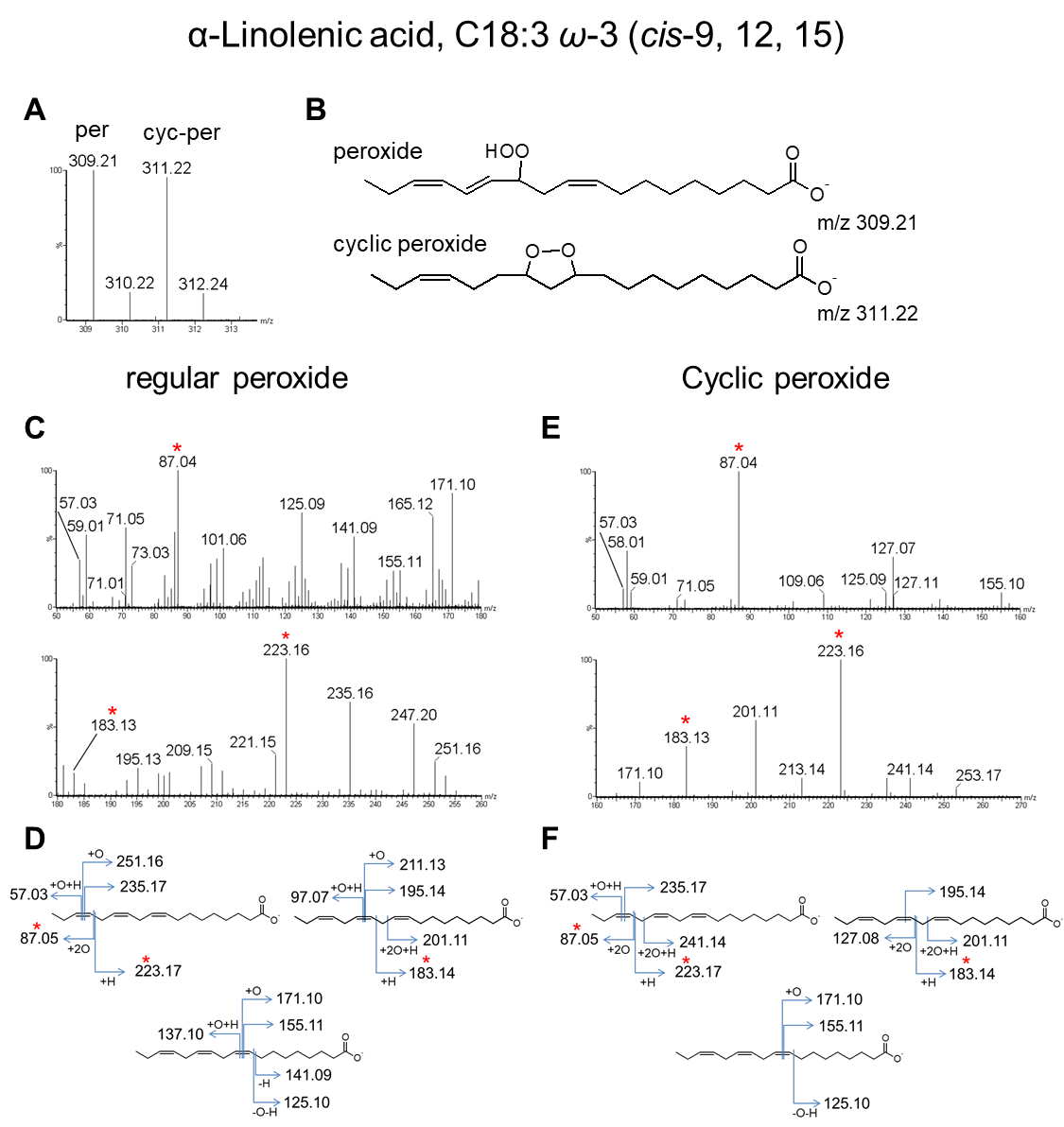
**

**Figure S6.** Detailed fragmentation pattern of two types of peroxide of α-linolenic acid. (A) Mass spectra of peroxide (per) and cyclic peroxide (cyc-per) on solvent plasmatization. (B) Structural examples of proposed peroxide and cyclic peroxide of α-linolenic acid are shown. (C, D) Tandem mass spectrum of regular peroxide of α-linolenic acid (C) and depicted fragmentation patterns associated with each double bond (D) are shown. (E, F) Tandem mass spectrum of cyclic peroxide of α-linolenic acid (E) and depicted fragmentation patterns (F) are shown. Diagnostic fragment ions are marked by asterisks.

**
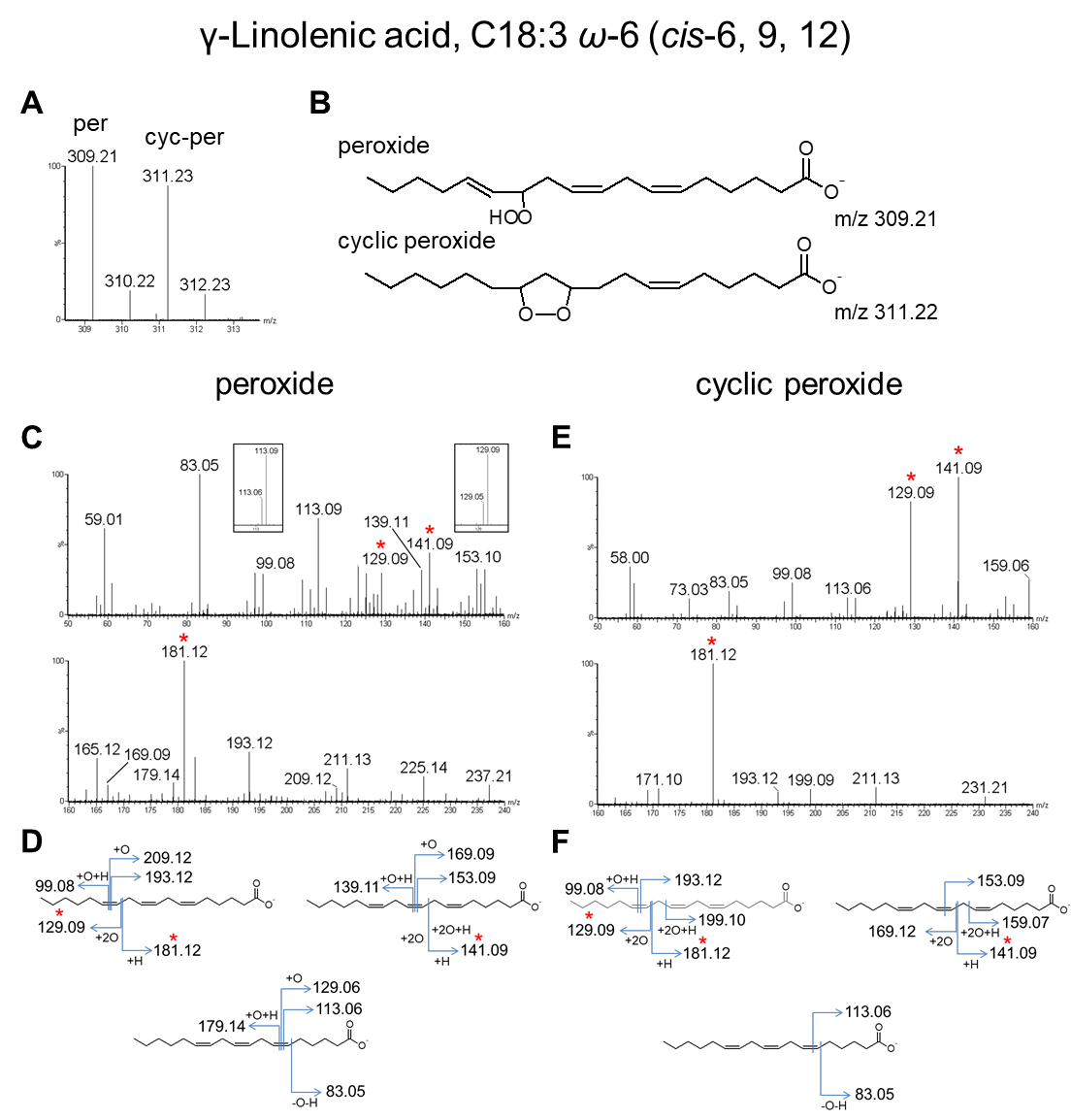
**

**Figure S7.** Detailed fragmentation pattern of two types of peroxide of γ-linolenic acid. (A) Mass spectra of peroxide (per) and cyclic peroxide (cyc-per) on solvent plasmatization. (B) Structural examples of proposed peroxide and cyclic peroxide of γ-linolenic acid are shown. (C, D) Tandem mass spectrum of regular peroxide of γ-linolenic acid (C) and depicted fragmentation patterns associated with each double bond (D) are shown. (E, F) Tandem mass spectrum of cyclic peroxide of γ-linolenic acid (E) and depicted fragmentation patterns (F) are shown. Diagnostic fragment ions are marked by asterisks.


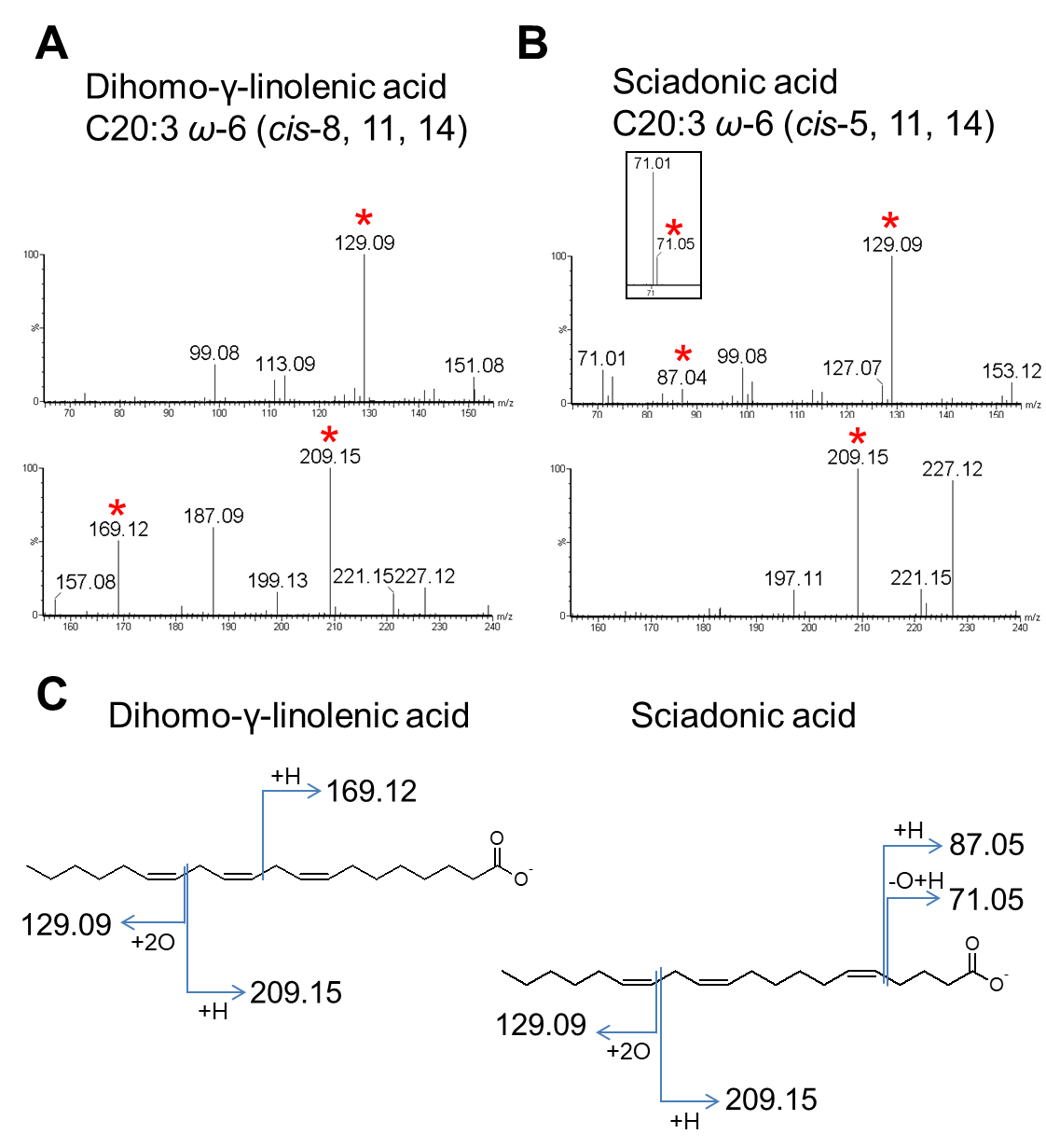
**Figure S8.** Structural determination of C20:3 FAs. (A, B) Fragmentation patterns of cyclic peroxide of Dihomo-γ-linolenic acid and sciadonic acid are shown. The diagnostic fragment ions are indicated by asterisks. It should be noted that common fragment ions originate from the identical double bonds while specific fragment ions originate from the distinct double bonds. (C) Graphical fragmentation patterns for C20:3 FAs are shown.

**
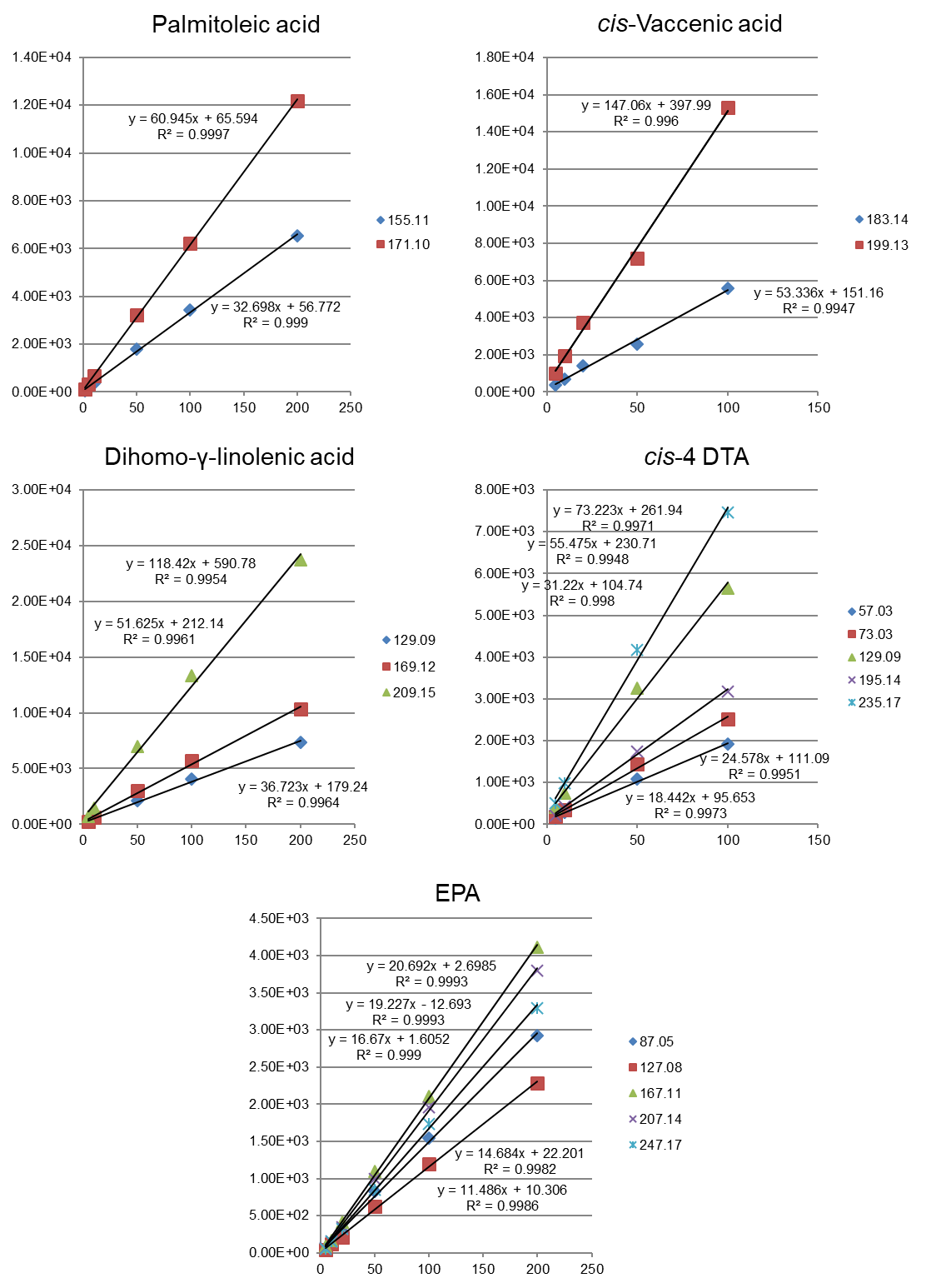
Figure S9.** Calibration curves for several FA species. Diagnostic fragment ions of MUFA, such as palmitoleic acid (C16:1 *cis*-9) and *cis*-vaccenic acid (C18:1 *cis*-11), as well as PUFA, dihomo-γ-linolenic acid (C20:3 *cis*-8, 11, 14), *cis*-4 DTA (C22:4 *cis*-4, 10, 13, 16), and EPA (C20:5 *cis*-5, 8, 11, 14, 17), were plotted individually. Calibration function and correlation coefficient (R^2^) are shown. The *x*-and *y*-axes represent FA concentration and intensity, respectively.

**
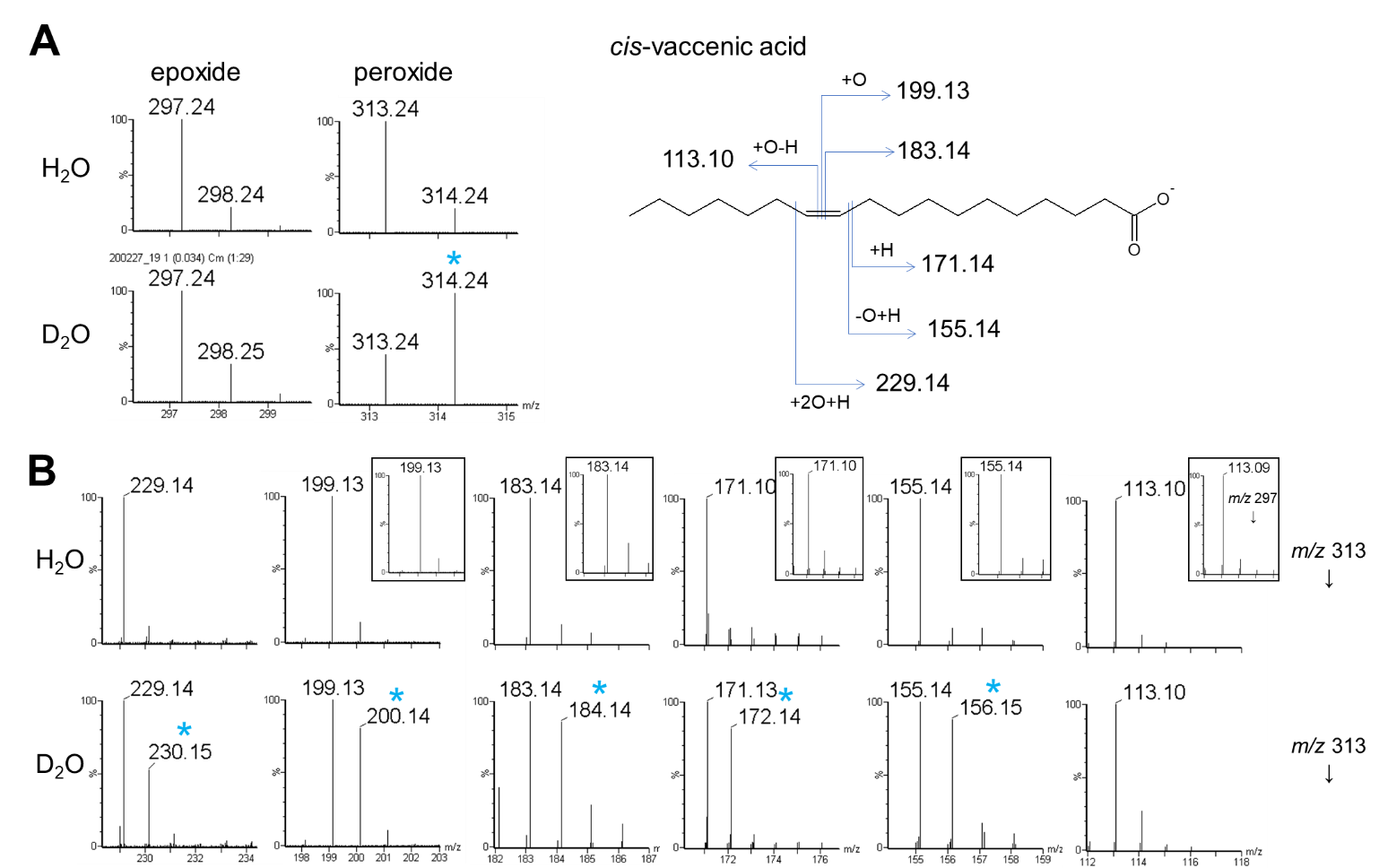
**

**Figure S10.** Fragmentation of *cis*-vaccenic acid with D_2_O. (A) Mass spectra of epoxide and peroxide of *cis*-vaccenic acid are shown. There is no increase in the mass of the epoxide produced with D_2_O as a solvent or regular water (H_2_O). There is a one-unit increase in the mass of the peroxide produced with D_2_O (asterisk). (B) Fragment ions of the peroxide of *cis*-vaccenic acid with D_2_O and H_2_O. The alpha fragment ions (*m/z* 229.14, 199.13, 183.14, 171.14, and 155.14) show mass increase by one unit (asterisks) when D_2_O is used as a solvent, in contrast to the omega fragment (*m/z* 113.10) that does not show clear mass increase. The mass increase of the alpha fragment ions is peroxide-specific, such that the equivalent fragments of epoxide with D_2_O (insets) do not show mass increase (the fragment ion with *m/z* 229.14 was not produced from epoxide).


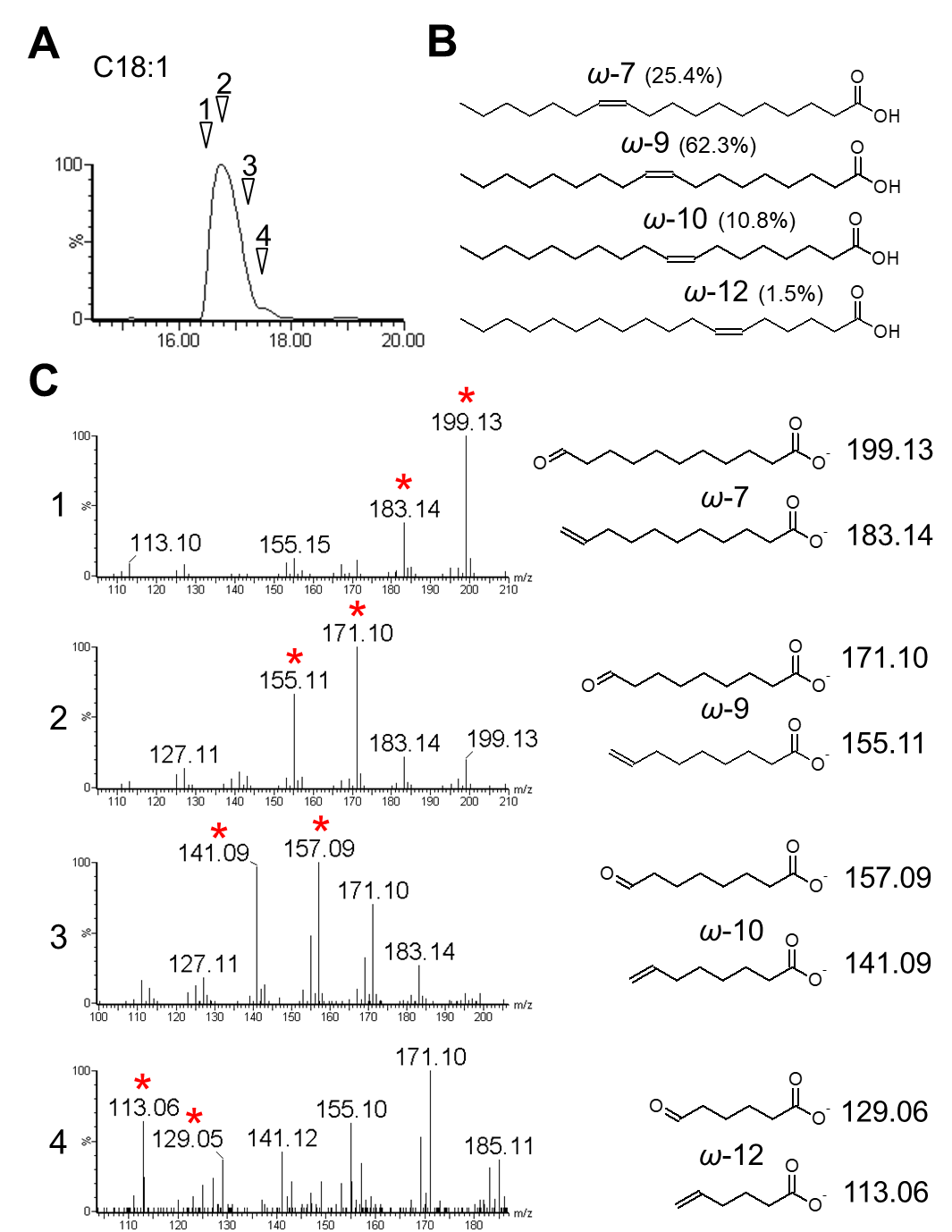


**Figure S11.** Identification of multiple C18:1 isomers from human fibroblasts. The same FA samples shown in Figure 6 were analyzed. (A) Mass chromatogram of C18:1 (*m/z* 281.25). Analytical time points where each isomer was detected are indicated. (B) A total of four C18:1 isomers each possessing double bonds at *ω*-7, *ω*-9, *ω*-10, and *ω*-12 were discovered, and their abundance ratio are shown. *cis/trans* isomerism is not considered. (C) Tandem mass spectra and structures of the diagnostic ions at the indicated time points in (A). The diagnostic fragment ions are indicated by asterisks.


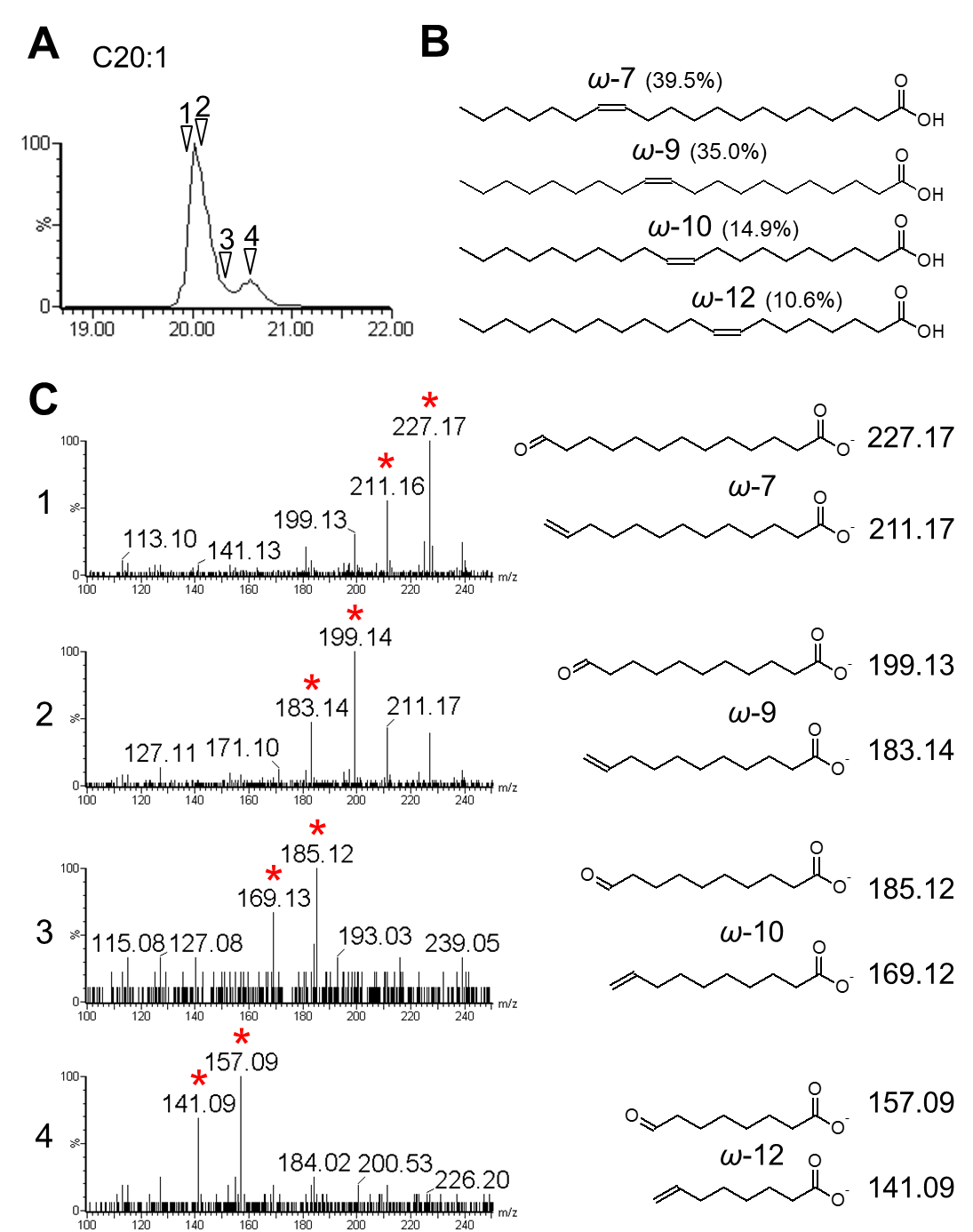


**Figure S12.** Identification of multiple C20:1 FA isomers from human fibroblasts. The same FA sample as shown in Figure 6 was analyzed. (A) Mass chromatogram of C20:1 on LC-ESI-MS (*m/z* 309.28). Analytical time points are indicated by arrowheads. (B) Four C20:1 isomers with double bonds either at *ω*-7, *ω*-9, *ω*-10, or *ω*-12 were detected and their abundance ratio were shown. *cis/trans* isomerism is not considered. (C) Tandem mass spectra and structures of the diagnostic ions at the indicated time points in (A) are shown. The diagnostic fragment ions are indicated by asterisks in the spectral graph.

**
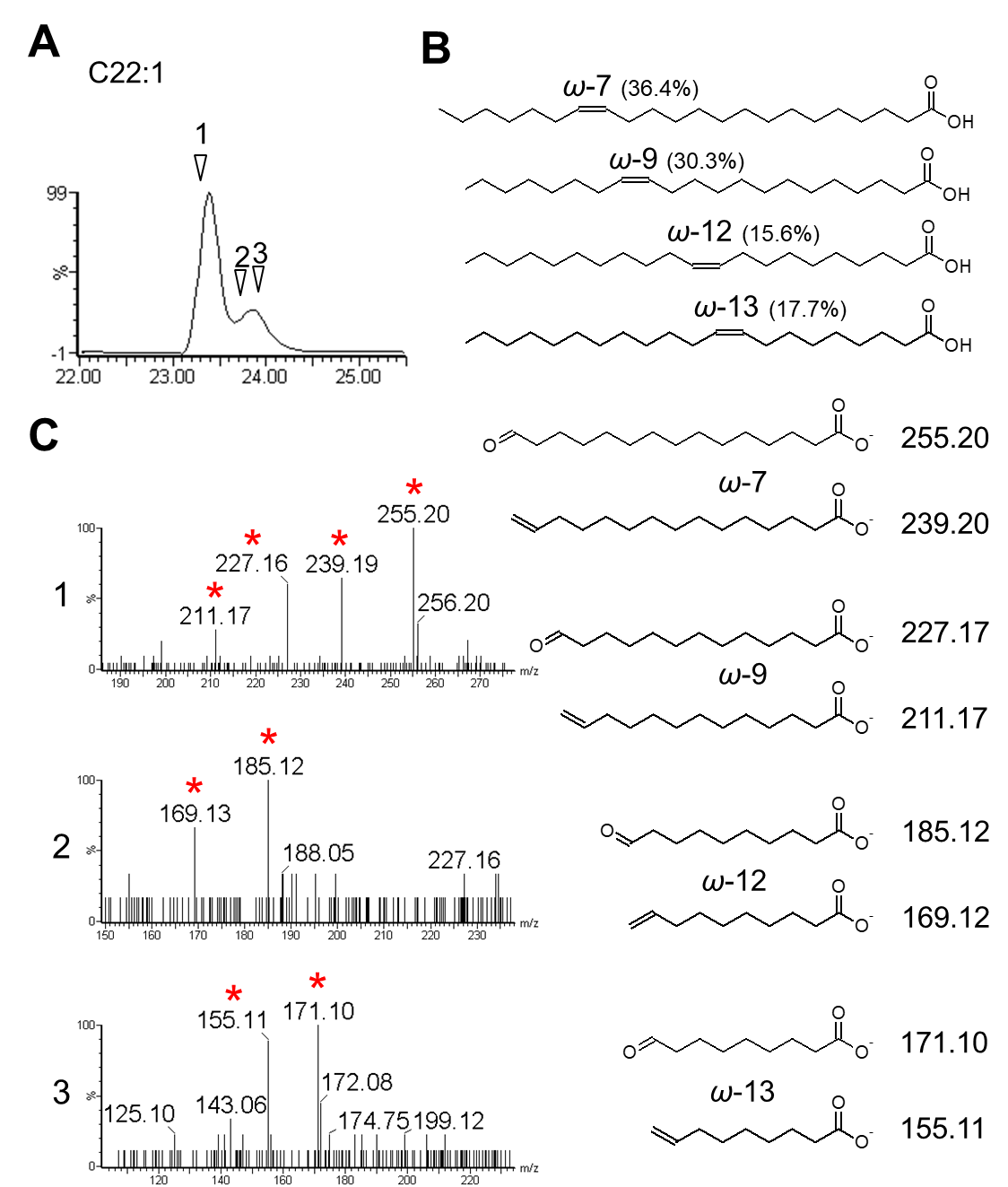
**

**Figure S13.** Identification of multiple C22:1 FA isomers from human fibroblasts. The same FA sample as shown in Figure 6 was analyzed. (A) Mass chromatogram of C22:1 on LC-ESI-MS (*m/z* 337.31). Analytical time points are indicated. (B) Four C22:1 isomers with double bond either at *ω*-7, *ω*-9, *ω*-12, or *ω*-13 position were detected and their abundance ratio were shown. *cis/trans* isomerism is not considered. (C) Tandem mass spectra and structures of the diagnostic ions at the indicated time points in (A) are shown. Diagnostic fragment ions are indicated by asterisks in the spectral graph.

**
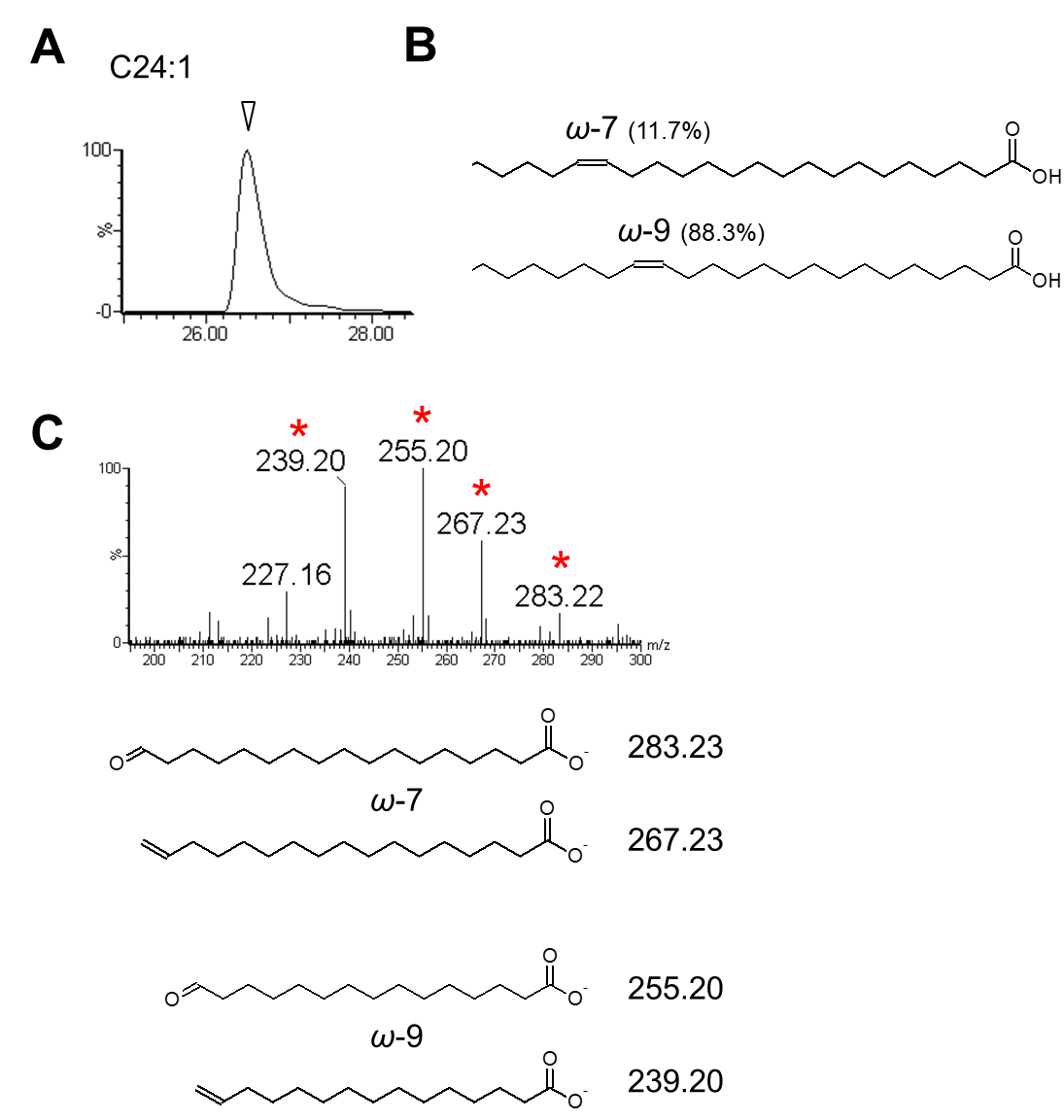
**

**Figure S14.** Identification of C24:1 FA isomers from human fibroblasts. The same FA sample as shown in Figure 6 was analyzed. (A) Mass chromatogram of C24:1 on LC-ESI-MS (*m/z* 365.34). The analytical time point is indicated by the arrowhead. (B) Two C24:1 isomers with double bonds either at *ω*-7 or *ω*-9 were discovered and their abundance ratio were shown. *cis/trans* isomerism is not considered. (C) Tandem mass spectra and structures of diagnostic ions at the indicated time point in (A) are shown. Diagnostic fragment ions are indicated by asterisks in the graph.

**
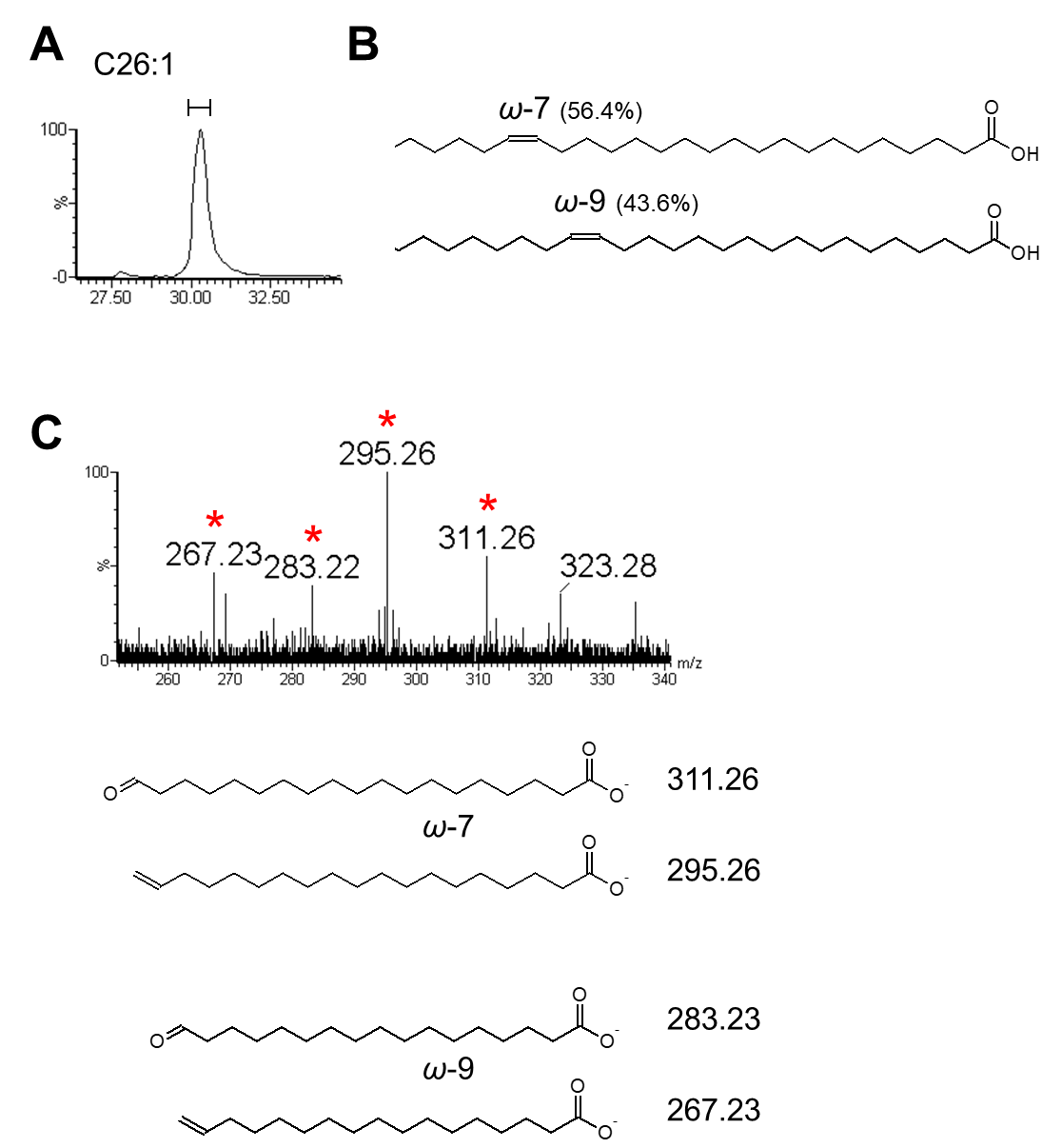
**

**Figure S15.** Identification of C26:1 FA isomers from human fibroblasts. The same FA sample as shown in Figure 6 was analyzed. (A) Mass chromatogram of C26:1 on LC-ESI-MS (*m/z* 393.37). Because of the low abundance of C26:1 species in the sample, the spectral data were combined throughout the peak, as indicated by the line. (B) Two C26:1 species with double bond either at *ω*-7 or *ω*-9 position were discovered and their abundance ratio were shown. *cis/trans* isomerism is not considered. (C) Tandem mass spectra and structures of the diagnostic ions are shown. Diagnostic fragment ions are indicated by asterisks in the graph.

**
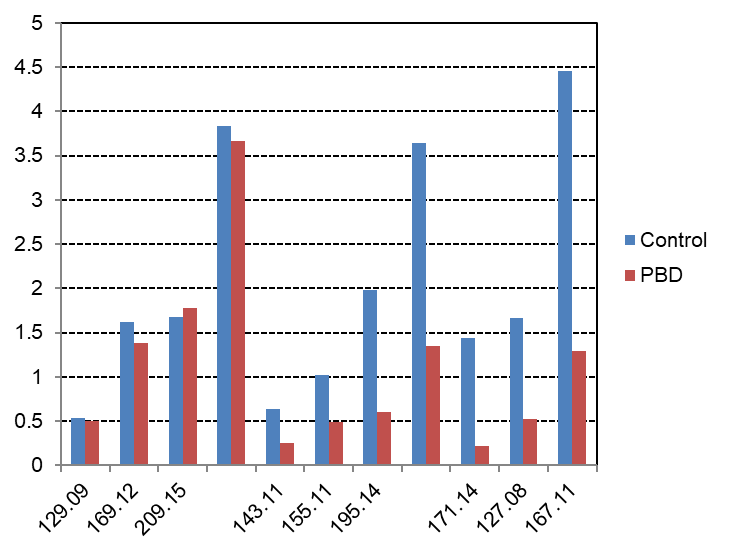
**

**Figure S16.** Comparison of C20:3 isomers found in human fibroblasts. Normalized intensities of diagnostic fragments from three C20:3 isomers against internal standard were compared (also see Figure 7). The blue bars represent wild-type fibroblasts and the red bars represent PBD fibroblasts. The numbers on the *x*-and *y*-axes indicate *m/z* values of diagnostic fragment ions and intensity, respectively.
